# Global, regional, and national burden of Pediatric and adolescent thyroid cancer from 1990 to 2021: a statistical analysis of prevalence, incidence, and DALYs

**DOI:** 10.3389/fonc.2025.1630648

**Published:** 2025-07-29

**Authors:** Rui Zhang, Zhenxin Mei, Shan Feng, Zongcun Chen

**Affiliations:** ^1^ Department of Endocrinology, The Second Affiliated Hospital of Hainan Medical University, Haikou, China; ^2^ Department of Oncology, The Second Affiliated Hospital of Hainan Medical University, Haikou, China; ^3^ Department of Emergency, The Second Affiliated Hospital of Hainan Medical University, Haikou, China

**Keywords:** pediatric and adolescent thyroid cancer, global, GBD, disease burden, frontier analysis, inequality analysis

## Abstract

**Background:**

Thyroid cancer is the most common malignant tumor of the endocrine system. Among children and adolescents, cancer ranks as the fourth leading cause of cancer-related deaths. Pediatric and adolescent thyroid cancer is a rare disease characterized by high metastatic and invasive properties. Currently, there remains a notable absence of comprehensive analyses addressing the global disease burden and evolving trends of Pediatric and adolescent thyroid cancer.

**Methods:**

This study used the Global Burden of Disease (GBD) database (1990-2021) to extract three key metrics: prevalence, incidence, and disability adjusted life years, for Pediatric and adolescent thyroid cancer cases. The data were shown as numerical counts and age-standardized counts per 100,000 within a population with 95% uncertainty intervals (UI). It assessed the evolving trends in the burden of thyroid cancer among children and adolescents by employing the EAPC (Estimated Annual Percentage Change) and percentage change metric.

**Results:**

In 2021, the global prevalence, incidence, and disability-adjusted life-years of pediatric and adolescent thyroid cancer reached 91,313 cases, 10,137 cases, and 60,185 years respectively. These figures show 94%, 91%, and 25.2% increases over 1990 figures. Over the 32-year observation period, all three metrics demonstrated sustained upward trajectories, with EAPC of 1.58 (95% UI: 1.48-1.68) for prevalence, 1.52 (95% UI: 1.42-1.62) for incidence, and 0.08 (95% UI: 0.00-0.15) for DALYs. In the 5 SDI regions, the prevalence and incidence are showing an upward trend, while DALYs rates showed divergent patterns: a marginal increase in low-middle SDI regions (EAPC=0.13, 95% UI: -0.07-0.32) contrasted with declining trends in other regions. A notable shift in disease burden has been observed across different regions, with high prevalence, incidence, and DALY rates moving from high-income to low-income countries. Age and sex-stratified analysis demonstrated that in 2021, the 20-24-year age group accounted for over 50% of global prevalence, incidence, and DALY rates, with females bearing a significantly higher disease burden than males. As for the global trend, there was a positive correlation between SDI and prevalence, but an inverse association between SDI and incidence, and an inverse relation between SDI and DALY rate. Decomposition analysis attributed 70.61% of prevalence changes and 69.9% of incidence variations to epidemiological factors, while population growth accounted for 71.83% of DALY rate alterations.

**Conclusion:**

Despite the declining prevalence, incidence, and DALY rates in high-income regions, the global disease burden of pediatric and adolescent thyroid cancer continues to rise, particularly among females and within the 20–24 age group. Therefore, implementing targeted interventions in countries or regions with a higher disease burden could contribute to reducing the global disease burden, optimizing resource allocation, and exploring diverse treatment modalities may be pivotal to addressing the issue.

## 1 Introduction

Thyroid carcinoma, the most common endocrine tumor, originates from thyroid follicular epithelial cells or parafollicular cells. Ranked as the 9th most frequently occurring cancer globally, it also shows obvious gender differences, being the 7th most common cancer in women (1). The World Health Organization classifies thyroid cancer as 4 main histopathologic subtypes: papillary, follicular, medullary, and anaplastic carcinoma (2). ​The incidence of thyroid cancer shows significant differences by region and gender. In middle- and high-income countries, the higher incidence of thyroid cancer may be associated with the extensive implementation of medical imaging technology in the late 19th century, which has resulted in a significant rise in thyroid cancer diagnoses in high-income regions, with women exhibiting approximately threefold higher susceptibility to the disease compared to men (3). Despite elevated incidence, thyroid cancer maintains a favorable prognosis with lower mortality rates and a 5-year relative survival rate of 98.6% (4).

Among pediatric populations, where cancer represents the fourth leading cause of mortality, Pediatric and adolescent thyroid cancer remains uncommon, with an annual incidence of 1–3 cases per million children. These pediatric cases frequently present with lymph node involvement and pulmonary metastases at diagnosis (5). Epidemiologic data indicate cumulative risk estimates of 12% for adolescents versus 2% for younger children. Even though the incidence of pediatric and adolescent thyroid cancer remains relatively rare, pediatric thyroid cancer tends to be much more aggressive compared to adult thyroid cancer; it has a strong tendency towards remote metastasis. And the number of people with thyroid cancer is still in an increase every year. Therefore, the World Health Organization has designated pediatric and adolescent thyroid cancer as an issue of public health and included it in the global burden of disease evaluation (6, 7).

The Global Burden of Disease (GBD) study systematically gathers and analyzes data on approximately 371 diseases, serving as a robust instrument for evaluating annual incidence, mortality, disability-adjusted life years, and associated risk factors (8, 9). Prior research has demonstrated that the incidence of thyroid cancer across all age groups is projected to increase in the future, with a particularly rapid growth rate observed among children and adolescents (10, 11). Therefore, this study, utilizing the latest GBD data, performed a comprehensive analysis of the incidence, prevalence, and disability-adjusted life years (DALYs) associated with thyroid cancer among children and adolescents from 1990 to 2021 at global, regional, and national levels, emphasizing temporal trends, age and gender distribution, disparities across varying socioeconomic development levels, inequality assessments, and frontier analyses, thereby furnishing crucial insights for devising more efficacious strategies and preventive interventions targeting thyroid cancer in this demographic.

## 2 Methods

### 2.1 Overview and data source

The GBD 2021 dataset encompasses estimates from 1990 to 2021 across 204 countries and 21 regions globally. It assesses 371 diseases and injuries across multiple metrics, including incidence, prevalence, disability-adjusted life years (DALYs), and the Socio-demographic Index (SDI). Among these, DALYs constitute a pivotal metric of disease burden, denoting the aggregate number of healthy life years lost from disease onset to mortality. This metric is calculated by summing Years of Life Lost due to premature mortality (YLLs) and Years Lived with Disability (YLDs) (8, 12), as defined by the formulae (Equations 1– 3):


(1) $$\begin{array}{c} YLLs=Number\;of\;deaths\;\times \;Standard\;life\;expectancy\;at\;age\;of\;death\; \end{array}$$

(2) $$\begin{array}{c} YLDs=Number\;of\;incident\;cases\;\times Disease\;weight\times Average\;duration\;of\;the\;condition \end{array}$$

(3) $$\begin{array}{c} YLDs=Number\;of\;prevalent\;cases\;\times Disease\;weight \end{array}$$

Researchers analyzed the global burden of pediatric and adolescent thyroid cancer in the GBD database using the following search strategies: The three indicators of prevalence, incidence, and DALYs were selected as metrics to assess the disease burden. The measurement standards were set as “cases and rates.” The time span was from 1990 to 2021. The age groups were described according to different stratifications. The regions covered 204 countries and territories. Additionally, the disease burden was analyzed across multiple geographic dimensions. Moreover, this study also conducted in-depth analyses of pediatric and adolescent thyroid cancer, including decomposition analysis, inequality analysis, and frontier analysis. Decomposition analysis aimed to identify distinct factors influencing health metrics and quantify their contributions. Inequity analysis examined health disparities among socioeconomically diverse populations to detect inequitable resource allocation. Frontier analysis identified potential improvement areas and gaps between nations with varying socioeconomic levels (13).

### 2.2 Socio−demographic index

The Socio-demographic Index (SDI) is a composite metric that evaluates the socioeconomic development status of nations or regions and correlates closely with health outcomes. This index incorporates three key components: the total fertility rate for women under 25 years of age, the average educational attainment of the population aged 15 years and above, and the lag-distributed income per capita (14). SDI values span from 0 (indicating the lowest level of development) to 1 (representing the highest level of development), with higher values denoting advanced socioeconomic conditions marked by enhanced educational attainment, elevated income levels, and diminished fertility rates (8). This study categorizes countries or regions into five SDI quintiles (low, low-middle, middle, high-middle, and high) to systematically investigate associations between pediatric and adolescent thyroid cancer epidemiology and socioeconomic development.

### 2.3 Sstatistics analysis

This study utilized statistical data from the GBD database to assess the disease burden of pediatric and adolescent thyroid cancer, with prevalence, incidence, and disability-adjusted life years (DALYs) rates as key metrics. Age-standardized rates per 100,000 population and their corresponding 95% uncertainty intervals (UI) were calculated. Temporal trends in these metrics were assessed through the calculation of the EAPC (Estimated Annual Percentage Change). An upward trend was defined as an EAPC estimate and 95% confidence interval (CI) both exceeding 0, a downward trend as both below 0, and a stable trend as an EAPC estimate and 95% CI inclusive of 0 (15, 16). All analyses were conducted utilizing R software version 4.3.3, with statistical significance defined as p < 0.05. The data utilized in this study were derived from the GBD database, which has received approval from the University of Washington Institutional Review Board and is publicly available.

## 3 Results

### 3.1 Global level

**Figure 1:**
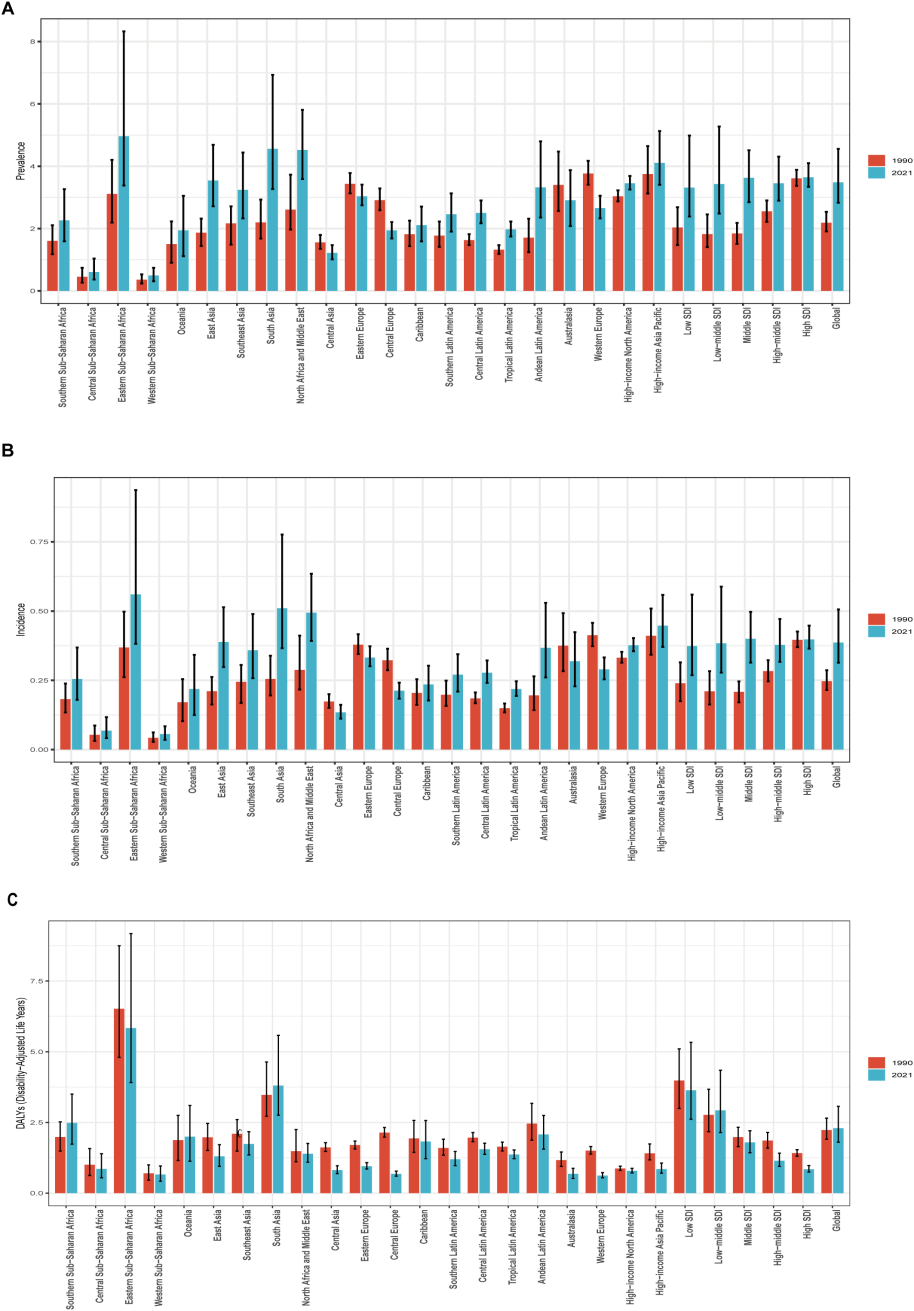
Prevalence, Incidence, and Disability-Adjusted Life-Years (DALYs) Rates for Pediatric and adolescent thyroid cancer From 1990 to 2021. **(A)** Prevalence rate. **(B)** Incidence rate. **(C)** DALYs rate.

The number of global pediatric and adolescent thyroid cancer cases increased from 47,131 (95% uncertainty interval [UI]: 41,137-4,707) in 1990 to 91,313 (95% UI: 74,182-119,506) in 2021, representing a 94% increase. Incident cases rose from 5,318 (95% UI: 4,641-6,181) in 1990 to 10,137 (95% UI: 8,228-13,275) in 2021, reflecting a 91% increase. DALYs increased from 48,068 (95% UI: 41,044-57,107) in 1990 to 60,185 (95% UI: 47,168-80,484) in 2021, with a 25.2% rise. In 1990, the global prevalence rate was 2.19 cases per 100,000 population (95% UI: 1.91-2.54), the incidence rate was 0.25 per 100,000 (95% UI: 0.22-0.29), and the DALYs rate was 2.23 per 100,000 (95% UI: 1.91-2.65). By 2021, the prevalence rate increased to 3.48 cases per 100,000 population (95% UI: 2.83-4.56), the incidence rate rose to 0.39 per 100,000 (95% UI: 0.31-0.51), and the DALYs rate reached 2.30 per 100,000 (95% UI: 1.80-3.07). From 1990 to 2021, global pediatric and adolescent thyroid cancer prevalence, incidence, and DALYs rates exhibited upward trends, with estimated annual percentage changes (EAPC) of 1.58 (95% UI: 1.48-1.68), 1.52 (95% UI: 1.42-1.62), and 0.08 (95% UI: 0.00-0.15), respectively (**Table 1**, **Supplementary Tables S1**, **S2**, **Figure 1**). These results indicate a continued increase in the global burden of pediatric and adolescent thyroid cancer.

**Table 1:** Prevalence of thyroid cancer in children and adolescents between 1990 and 2021 at the global and regional level.

Location	Prevalent cases			Prevalence rate		
	1990 (95% UI)	2021 (95% UI)	percentage change (100%)	1990 (95% UI)	2021 (95% UI)	EAPC (95% CI)
Global	47130.66 (41137.49,54707.45)	91312.80 (74181.97,119506.04)	0.94	2.19 (1.91,2.54)	3.48 (2.83,4.56)	1.58 (1.48,1.68)
High-middle SDI	9964.87 (8676.28,11336.07)	10899.49 (9153.09,13611.97)	0.09	2.55 (2.22,2.90)	3.45 (2.90,4.31)	1.21 (1.00,1.42)
High SDI	9916.71 (9256.86,10678.65)	9741.01 (8918.17,10976.70)	-0.02	3.61 (3.37,3.89)	3.65 (3.34,4.10)	0.19 (-0.02,0.4)
Low-middle SDI	8952.23 (6918.41,12050.71)	26374.75 (19081.02,40638.07)	1.95	1.82 (1.41,2.46)	3.43 (2.48,5.27)	2.00 (1.87,2.13)
Low SDI	4214 (3051.80,5564.98)	16362.77 (11802.96,24547.23)	2.88	2.03 (1.47,2.69)	3.31 (2.39,4.98)	1.45 (1.39,1.52)
Middle SDI	14038.89 (11494.58,16626.98)	27880.42 (21881.31,34646.53)	0.99	1.84 (1.51,2.18)	3.63 (2.85,4.51)	2.42 (2.28,2.56)
East Asia	9320.36 (7251.60,11647.71)	12045.01 (9228.14,15946.45)	0.29	1.87 (1.44,2.32)	3.54 (2.72,4.69)	2.35 (2.11,2.58)
Southeast Asia	4402.39 (3022.03,5507.43)	7805.9 (5599.85,10708.08)	0.77	2.17 (1.49,2.71)	3.24 (2.33,4.44)	1.19 (1.12,1.26)
Oceania	43.76 (26.44,64.94)	109.36 (62.63,171.70)	1.50	1.50 (0.90,2.23)	1.94 (1.11,3.05)	0.66 (0.48,0.84)
Central Asia	429.33 (372.50,495.85)	376.04 (313.32,453.36)	-0.12	1.56 (1.35,1.80)	1.22 (1.02,1.47)	-0.57 (-1.5,0.36)
Central Europe	1130.69 (1006.66,1275.3)	481.13 (415.94,547.31)	-0.57	2.91 (2.59,3.29)	1.94 (1.68,2.21)	-1.17 (-1.39,-0.95)
Eastern Europe	2245.44 (2045.88,2472.86)	1373.67 (1245.07,1542.82)	-0.39	3.44 (3.13,3.78)	3.03 (2.75,3.41)	0.19 (-0.40,0.78)
High-income Asia Pacific	2133.61 (1776.36,2650.05)	1562.63 (1290.8,1960.49)	-0.27	3.75 (3.12,4.65)	4.11 (3.40,5.13)	0.10 (-0.31,0.51)
Australasia	234.88 (176.91,309.05)	241.3 (172.62,320.93)	0.03	3.40 (2.56,4.47)	2.91 (2.08,3.87)	0.09 (-0.75,0.94)
Western Europe	4399.26 (3977.15,4882.22)	2703.42 (2367.84,3110.15)	-0.39	3.76 (3.41,4.17)	2.65 (2.33,3.05)	-0.87 (-1.35,-0.38)
Southern Latin America	323.12 (257.65,405.64)	532.41 (410.65,678.88)	0.65	1.77 (1.41,2.23)	2.46 (1.90,3.13)	1.29 (1.06,1.52)
High-income North America	2614.1 (2470.16,2782.56)	3475.25 (3277.27,3712.03)	0.33	3.03 (2.87,3.23)	3.45 (3.25,3.69)	0.46 (0.33,0.59)
Caribbean	268.12 (212.28,332.65)	332.18 (250.96,425.57)	0.24	1.82 (1.44,2.26)	2.11 (1.59,2.71)	0.64 (0.46,0.82)
Andean Latin America	287.37 (209.13,389.24)	807.15 (570.82,1168.04)	1.81	1.71 (1.24,2.32)	3.32 (2.35,4.80)	2.38 (2.08,2.69)
Central Latin America	1211.77 (1096.67,1353.97)	2257.57 (1957.45,2619.49)	0.86	1.63 (1.47,1.82)	2.50 (2.17,2.90)	1.54 (1.38,1.69)
Tropical Latin America	875.76 (786.16,973.27)	1400.31 (1242.24,1576.15)	0.60	1.32 (1.19,1.47)	1.97 (1.74,2.23)	1.50 (1.11,1.88)
North Africa and Middle East	3902.61 (2943.44,5603.25)	10136.06 (8042.56,13014.32)	1.60	2.61 (1.97,3.73)	4.52 (3.59,5.81)	2.43 (2.17,2.69)
South Asia	9989.06 (7617.83,13311.66)	33880.42 (24300.77,51636.86)	2.39	2.20 (1.68,2.93)	4.56 (3.27,6.93)	2.35 (2.18,2.51)
Central Sub-Saharan Africa	109.61 (63.72,179.04)	371.08 (225.75,631.53)	2.39	0.45 (0.26,0.74)	0.60 (0.36,1.04)	1.17 (0.87,1.47)
Eastern Sub-Saharan Africa	2528.88 (1788.12,3414.17)	9640.62 (6578.23,16130.72)	2.81	3.11 (2.19,4.21)	4.96 (3.38,8.33)	1.34 (1.20,1.47)
Southern Sub-Saharan Africa	377.52 (277.03,495.52)	679.38 (477.55,979.69)	0.80	1.60 (1.18,2.11)	2.26 (1.59,3.26)	1.23 (0.74,1.72)
Western Sub-Saharan Africa	303.01 (199.22,442.24)	1101.92 (688.34,1648.30)	2.64	0.36 (0.24,0.53)	0.50 (0.31,0.74)	1.20 (1.09,1.32)

### 3.2 SDI regional level

**Figure 2:**
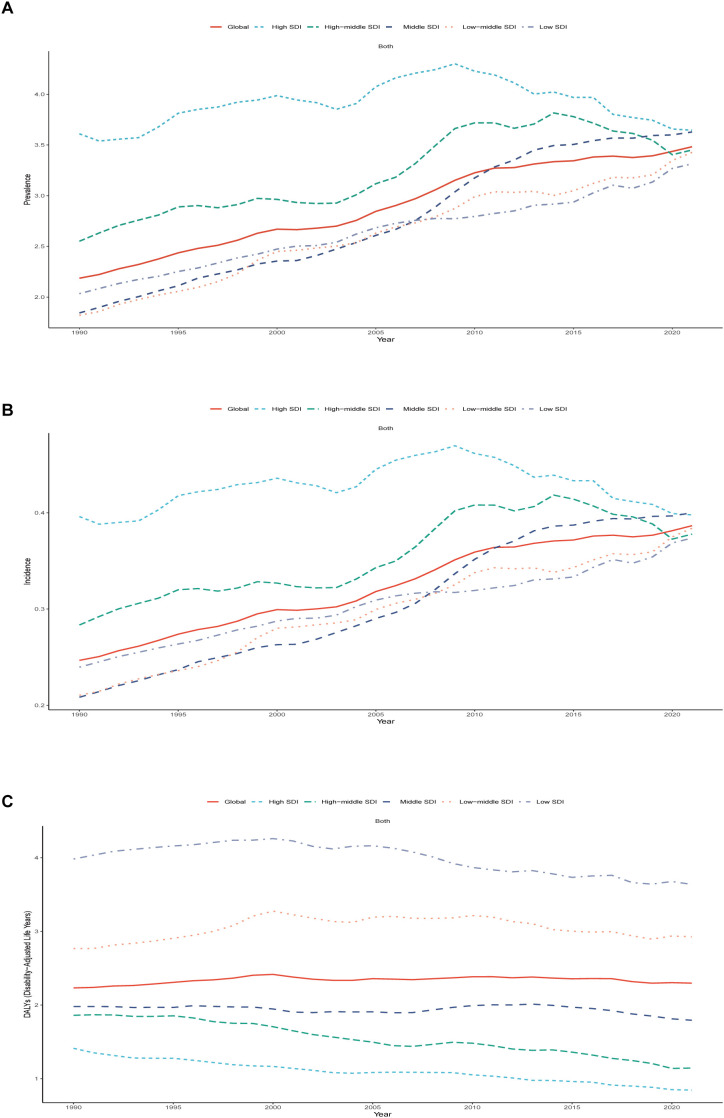
Epidemiologic Trends of Prevalence, Incidence, and Disability-Adjusted Life-Years (DALYs) Rates in 5 Sociodemographic Index (SDI) Regions of Pediatric and Adolescent Thyroid Cancer From 1990 to 2021. **(A)** Trend in prevalence rate. **(B)** Trends in incidence rate. **(C)** Trends in DALYs rate.

In 1990, the Middle SDI region had the highest number of prevalent cases (14,039; 95% UI: 11,495-16,627), incident cases (1,587; 95% UI: 1,302-1,876), and DALYs (15,000; 95% UI: 12,489-17,636) for pediatric and adolescent thyroid cancer. By 2021, this region maintained the highest prevalent cases (27,880; 95% UI: 21,881-34,647) and incident cases (3,074; 95% UI: 2,415-3,818), while the highest DALYs shifted to the Low-middle SDI region (22,489; 95% UI: 16,469-33,441). In 2021, High SDI regions exhibited the highest prevalence rate of pediatric and adolescent thyroid cancer, while High and Middle SDI regions jointly showed the highest incidence rate, and Low SDI regions recorded the highest DALYs rate. The Low SDI region demonstrated the largest absolute increases in prevalence, incidence, and DALYs from 1990 to 2021. In contrast, the High SDI region experienced a 2% reduction in both prevalent and incident case counts compared to 1990, indicating a decline in absolute case numbers by 2021. However, age-standardized trend analysis from 1990 to 2021 revealed persistent upward trajectories in High SDI regions, with estimated annual percentage changes (EAPC) of 0.19 (95% UI: -0.02-0.40) for prevalence and 0.17 (95% UI: -0.04-0.38) for incidence. All five SDI regions demonstrated increasing trends in prevalence and incidence rates during this period, with the most significant growth observed in the Middle SDI region. For DALYs, only the Low-middle SDI region exhibited an upward trend (EAPC = 0.13; 95% UI: -0.07-0.32), while other regions displayed downward trends (negative EAPC values). (**Table 1**, **Supplementary Tables S1**, **S2**, **Figures 1**, **2**).

### 3.3 Geographic regional level

**Figure 3:**
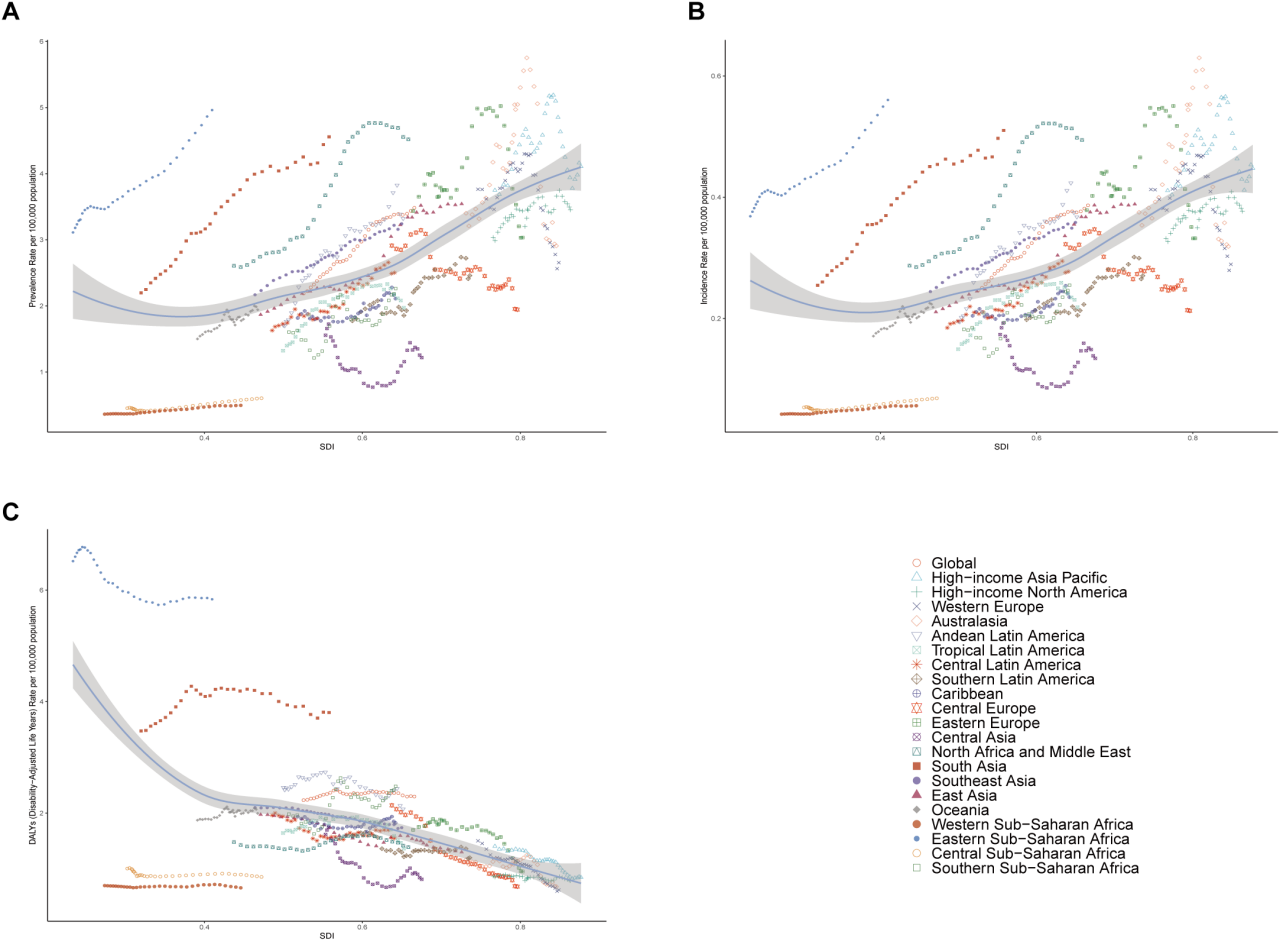
Prevalence, Incidence, and Disability-Adjusted Life-Years (DALYs) Rates in global and 21 geographic Regions of Pediatric and adolescent thyroid cancer from 1990 to 2021. **(A)** Prevalence rate. **(B)** Incidence rate. **(C)** DALYs rate.

Among the 21 geographical regions, most demonstrated increasing trends in pediatric and adolescent thyroid cancer prevalence and incidence over time, with declines observed only in Central Asia, Western Europe, and Central Europe. South Asia consistently exhibited the highest absolute disease burden during the 32 years: prevalent cases surged from 9,989 (95% UI: 7,618-13,312) in 1990 to 33,880 (95% UI: 24,301-51,637) in 2021; incident cases rose from 1,158 (95% UI: 887-1,540) to 3,793 (95% UI: 2,724-5,782); and DALYs increased from 15,866 (95% UI: 12,395-21,169) to 28,165 (95% UI: 20,437-41,389). Oceania maintained the lowest case counts for both prevalence and incidence. Notably, the highest prevalence shifted from Western Europe (3.76 per 100,000; 95% UI: 3.41-4.17) in 1990 to Eastern Sub-Saharan Africa (4.96 per 100,000; 95% UI: 3.38-8.33) by 2021. A parallel transition occurred for incidence rates, with Western Europe’s leading position (0.41 per 100,000; 95% UI: 0.37-0.46) overtaken by Eastern Sub-Saharan Africa (0.56 per 100,000; 95% UI: 0.38-0.94) in 2021. Western Europe exhibited declining trends in both prevalence (EAPC: -0.87; 95% UI: -1.35-0.38) and incidence (EAPC: -0.89; 95% UI: -1.36-0.41). Throughout the period from 1990 to 2021, Eastern Sub-Saharan Africa consistently demonstrated the highest DALYs rate for pediatric and adolescent thyroid cancer, whereas Western Sub-Saharan Africa consistently exhibited the lowest DALYs rate. A predominant downward trend in DALYs was observed across most regions, with only select areas demonstrating increases. The most substantial decline occurred in Central Europe (EAPC: -3.56; 95% CI: -3.84–3.28), whereas Southern Sub-Saharan Africa experienced the most pronounced increase (EAPC: 0.89; 95% CI: 0.48-1.30) (**Table 1**, **Supplementary Tables S1**, **S2**, **Figure 1**, **3**, **Supplementary Figures S1**-**S3**). These patterns indicate a progressive epidemiological transition, characterized by the shifting of prevalence and incidence rates from high- and upper-middle-income regions to low-income areas. This trend is likely associated with socioeconomic development, enhanced health education initiatives in high-income regions, and the progressive dissemination of medical technologies to low-income settings.

### 3.4 National level

**Figure 4:**
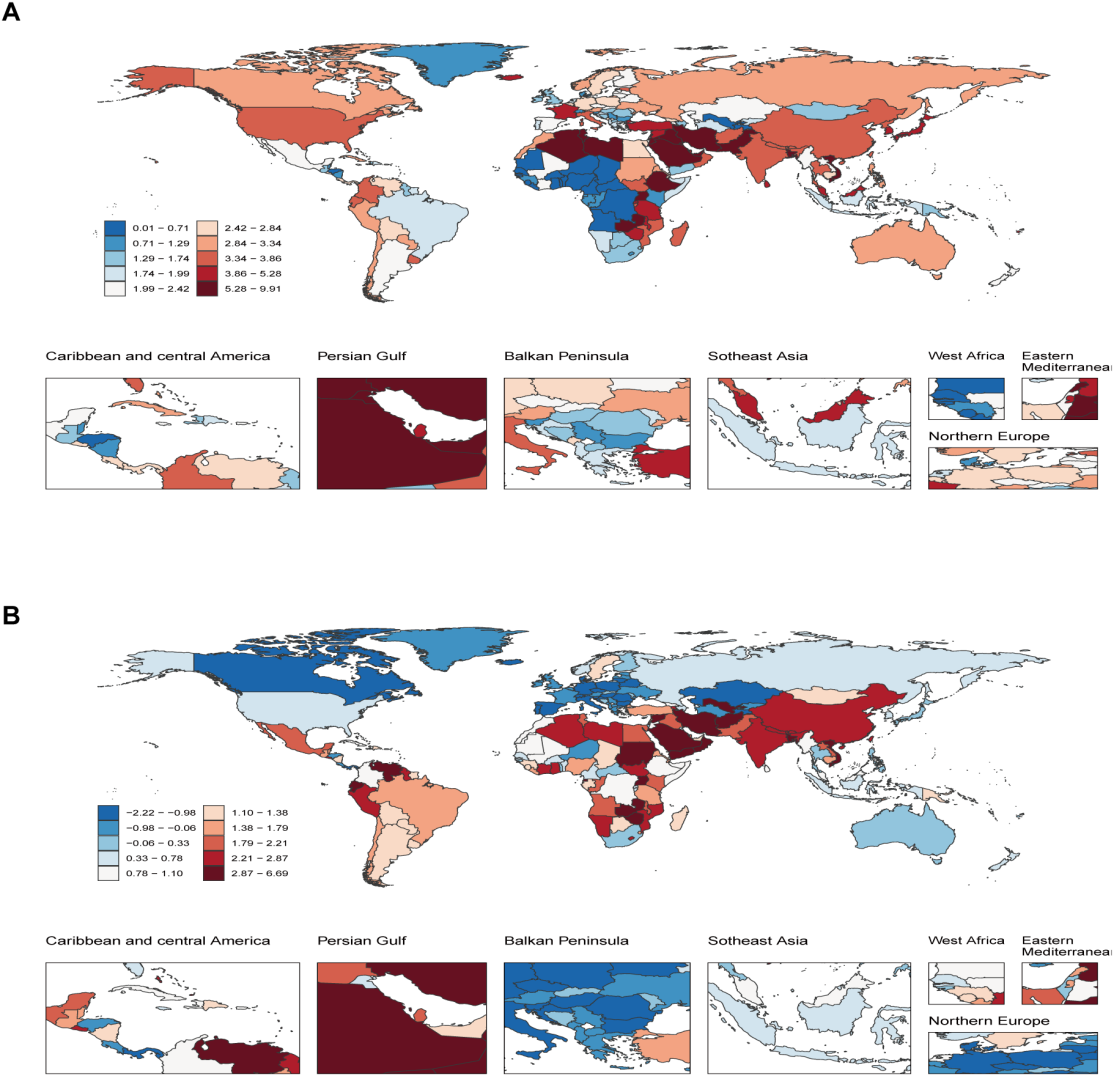
Temporal trend of Pediatric and adolescent thyroid cancer globally. **(A)** Percentage change in prevalent cases across 204 countries in 1990 and 2021. **(B)** EAPC in prevalence rates across 204 countries from 1990 to 2021. EAPC, Estimated Annual Percentage Change.

In 2021, global mapping revealed that regions with high prevalence and incidence rates of pediatric and adolescent thyroid cancer are mainly distributed in Asia, the Persian Gulf, some areas of Southeast Asia, and the Middle East and North Africa. Regions with low incidence rates were mostly distributed in some areas of Europe, West Africa, and some regions in South America. DALYs rates were elevated in South Asia, Southeast Asia, the Persian Gulf, Northern and Eastern Africa, and South America, whereas lower DALYs rates were recorded in regions such as North America, Northern Europe, and Oceania. From 1990 to 2021, as observed from the EAPC map, most countries had rising trends in prevalence and incidence rate, especially in Asia, Africa, South America, and the Persian Gulf. In contrast, Northern Europe, Canada, and the Balkan Peninsula exhibited significant declines. Conversely, DALYs rates demonstrated downward trends in most countries, though increases persisted in parts of Africa, South America, and the Persian Gulf. Among 204 nations, Cabo Verde showed the most substantial increases in prevalence, incidence, and DALYs rates, with EAPC values of 6.63 (95% UI: 5.50-7.77), 6.54 (95% UI: 5.40-7.70), and 4.43 (95% UI: 2.75-6.14), respectively. Luxembourg showed the largest EAPC in terms of decline in prevalence and incidence (EAPC: -2.20 [95% UI: -2.79–1.60] and -2.23 [95% UI: -2.82–1.63]), whereas Switzerland experienced the most significant reduction in DALYs (EAPC: -4.90 [95% UI: -5.39–4.41]) (**Supplementary Table S3**, **Figure 4**, **Supplementary Figures S4**, **S5**). ​These results suggest that the prevalence, incidence and DALY rates of pediatric and adolescent thyroid cancer are related to the development level and allocation of health care resources, and the high-income countries will see a gradual decline in prevalence, incidence and DALY rates over time, whereas Low-income countries see an increase.

### 3.5 Age patterns and sex patterns

**Figure 5:**
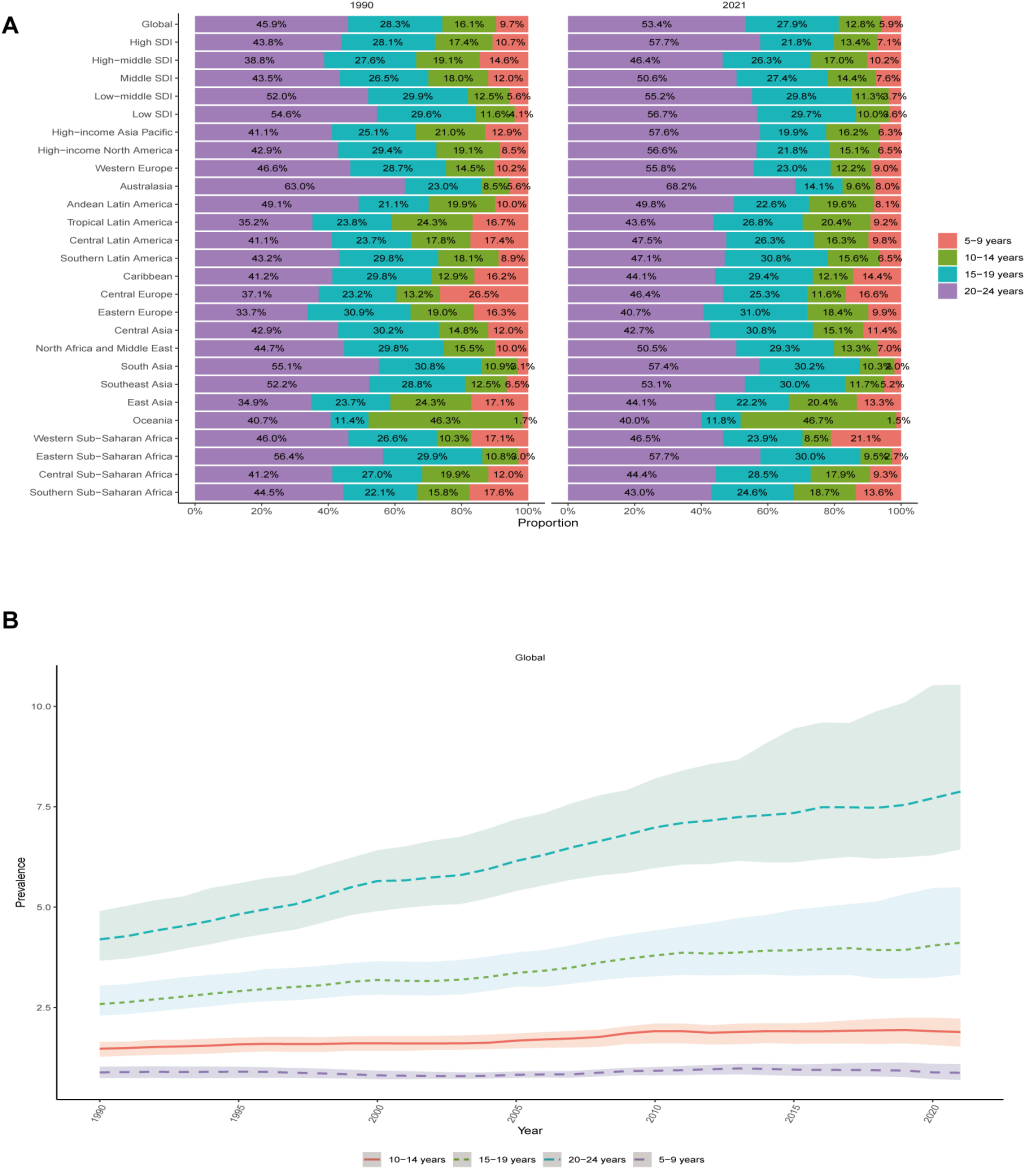
Temporal trend of Pediatric and adolescent thyroid cancer by age pattern at the Global Level. **(A)** The distribution of prevalence rates across 4 age groups as percentages globally, in 5 territories, and 21 GBD regions in 1990 and 2021. **(B)** Trends of Prevalence Rates in 4 age groups of Pediatric and Adolescent Thyroid Cancer From 1990 to 2021.

From 1990 to 2021, the prevalence and incidence of thyroid cancer among children and adolescents exhibited sustained growth in the 15–19 and 20–24 age groups, a modest rise in the 10–14 age group, and relative stability in the 5–9 age group. Notably, the incidence in the 20–24 age group remained consistently high throughout 2010–2020, plateauing at approximately 4,000 cases per 100,000 population. DALYs ratios showed a minor upward trend in the 20–24 group, stability in the 15–19 group, and minimal drops in the 5–9 and 10–14 groups. Age distribution patterns revealed that by 2021, the 20–24 age group accounted for over 50% of global thyroid cancer prevalence, incidence, and DALYs rates across all age groups combined. Most regions demonstrated increasing rates with age; however, exceptions were observed. For instance, Oceania reported the highest rates in the 10–14 age group (46.7% prevalence, 46.8% incidence, and 54.5% DALYs in 2021), while Western Sub-Saharan Africa recorded the lowest (8.5% prevalence, 8.5% incidence, and 8.7% DALYs) (**Supplementary Tables S4**-**S6**, **Figure 5**, **Supplementary Figures S6**, **S7**). The 2021 gender distribution chart demonstrates that the prevalence and incidence rates of pediatric and adolescent thyroid cancer are consistently higher among females compared to males on a global scale and across most regions. In Eastern Sub-Saharan Africa and Low-middle SDI regions, the rate for females is about three times that of males. Only in a few regions, such as Australasia and Western Europe, do males have a higher prevalence and incidence rate than females. At the same time, the gender difference in the DALYs rate for Pediatric and adolescent thyroid cancer is not significant, but globally, the DALYs rate for females is still higher than that for males. (**Supplementary Tables S7**-**S9**, **Supplementary Figure S8**). These findings indicate that among children and adolescents, thyroid cancer prevalence, incidence, and DALYs rates are more common in females than in males. Furthermore, the age group of 20–24 years bears the most significant global and regional burden of thyroid cancer, as evidenced by the persistent upward trajectory in prevalence, incidence, and DALYs rates over time.

### 3.6 The association between Pediatric and adolescent thyroid cancer and SDI

In terms of overall trends, the prevalence and incidence of pediatric and adolescent thyroid cancer demonstrated a positive correlation with the Socio-demographic Index (SDI), showing a declining trend when SDI was below 0.3. However, as economic development progressed beyond this threshold, the disease burden gradually shifted to an increasing trend. The upper-right quadrant highlights regions characterized by both high socioeconomic status and increased prevalence and incidence rates, such as Australasia, High-income Asia Pacific, and Eastern Europe. Conversely, DALYs exhibited a negative correlation with SDI, where higher SDI regions consistently demonstrated lower DALYs burdens. Eastern Sub-Saharan Africa represented a characteristic low-SDI, high-DALYs region. Most areas clustered within the SDI range of 0.4-0.8 showed a slowly declining disease burden trend. Among the 204 countries analyzed, those with SDI values ranging from 0.7 to 0.9 demonstrated relatively stable burdens in terms of the prevalence and incidence rates of pediatric and adolescent thyroid cancer. However, certain regions exhibited disease burdens surpassing anticipated thresholds: Libya, Saudi Arabia, Niue, and Vietnam regarding prevalence and incidence rates, and Ethiopia, Pakistan, and Uganda concerning DALYs burdens. (**Figure 3**, **Supplementary Figure S9**).

### 3.7 Decomposition analysis

Over the past 32 years, the global prevalence, incidence, and DALYs rates of Pediatric and adolescent thyroid cancer have increased significantly. Epidemiological changes accounted for the largest proportion of the increases in both prevalence (70.61%) and incidence (69.9%), followed by population growth, while aging demonstrated the smallest impact. In contrast, population growth contributed most substantially to DALYs rates (71.83%). The most significant increases in both prevalence and incidence across the five SDI regions were particularly evident in the Low-middle SDI and Middle SDI regions. Aging exhibited a negative impact in High SDI, High-middle SDI, and Middle SDI regions. While the contributions of epidemiological changes and aging were comparable in Low SDI regions, the most prominent epidemiological changes were observed in Middle SDI and Low-middle SDI regions. Population growth played a relatively minor role in all regions except High SDI. For DALYs rate, low-middle SDI and low SDI showed an elevated proportion, the aging factor is the most striking (56.47% and 91.15%) On the contrary, it showed that High SDI, High-middle SDI and Middle SDI have had a great decline, only showing the population growth with positive effects and aging, epidemiological changes with negative effects. Regionally, South Asia had the largest increase in prevalence, incidence and DALYs rates. Especially epidemiological factors have had the highest impact on prevalence and incidence rates as compared to other factors, the aging was considered to be the most important factor in determining DALYs rates. Age distribution and epidemiological transitions in East Asia were the most essential influencing factors for regional health. While examining prevalence and incidence rates, it was observed that aging contributed negatively at -240.84%, in contrast to epidemiological changes which manifested a positive impact of 273.43%. Both aging and epidemiological transitions demonstrated adverse effects on DALY rates, reflecting complex health challenges. (**Supplementary Tables S10**-**S12**, **Supplementary Figure S10**).

### 3.8 Frontier analysis

Utilizing data spanning from 1990 to 2021, this study conducted a frontier analysis to assess the burden of thyroid cancer among pediatric and adolescent populations across various regions or countries with their socioeconomic development levels, while also examining these regions’ frontier positions and their potential for enhancing health burden reduction. The 15 countries with the greatest potential for improvement were: Eritrea, United Republic of Tanzania, Madagascar, Rwanda, Burundi, Malawi, Comoros, Niue, Mozambique, Zimbabwe, Zambia, Tokelau, Uganda, Pakistan, and Ethiopia (**Supplementary Table S13**, **Supplementary Figure S11**). With socioeconomic development, the effective disparities increased to some extent, indicating that countries or regions with higher SDI have better potential for improving disease burden.

### 3.9 Inequality analysis.

This study employed both the slope index of inequality (SII) and concentration index (CI) to examine the absolute and relative socioeconomic inequalities, respectively, in pediatric and adolescent thyroid cancer DALYs rates associated with SDI. SII analysis shows that compared to 1990, the SII gradient is greater in 2021, high-SDI regions lead to further decreases in DALYs rates for high-SDI countries, which exacerbates absolute disparities between high-SDI and low-SDI countries. CI result revealed persistent relative inequalities that were high; -0.11(95% CI: -0.18–0.04) in 1990 and -0.22(95% CI: -0.28–0.16) in 2021. (**Supplementary Table S14**, **S15**, **Supplementary Figure S12**).

## 4 Discussion

Thyroid cancer, a prevalent malignancy within the endocrine system, exhibits pronounced aggressiveness and metastatic potential among pediatric and adolescent populations (17). This disease has now emerged as a salient global public health concern, imposing a substantial burden of illness. Given the current scarcity of research on the disease burden of pediatric and adolescent thyroid cancer, this study provides a reliable reference for this field through statistical analysis and global assessment. The study systematically characterized the epidemiology of pediatric and adolescent thyroid cancer from 1990 to 2021 across global regions, 204 countries, and five SDI-based demographic strata, with detailed age and sex stratification. It also incorporates frontier and inequality analyses among different countries and regions. Research findings indicate that over the past three decades, the incidence, prevalence, and DALYs rates of thyroid cancer among children and adolescents globally have exhibited a continuous upward trend, with the disease burden consistently escalating. Therefore, this study comprehensively assessed the disease burden of pediatric and adolescent thyroid cancer and provided a scientific basis for the development of effective preventive interventions.

The 2021 global data revealed 91,313 prevalent cases, 10,137 incident cases, and 60,185 DALYs of pediatric and adolescent thyroid cancer, showing significant increases compared to 2019. Regional analyses demonstrated that Western Europe had the highest prevalence and incidence rates in 1990, but exhibited declining trends over time, with estimated annual percentage changes (EAPCs) of -0.87 (95% UI: -1.35–0.38) and -0.89 (95% UI: -1.36–0.41), respectively. Conversely, low-income countries experienced progressive increases in both prevalence and incidence rates during the same period. Most nations and regions showed decreasing DALY rates, a phenomenon potentially associated with socioeconomic development in high-SDI areas. The rapid advancement of medical technology, particularly the widespread application of ultrasonography, has enabled precise detection of previously undiagnosed thyroid micronodules (18, 19). Therefore, the detection rate of thyroid cancer has significantly increased, which to some extent has driven the rise in incidence.

Age and sex factors may significantly influence the distribution of thyroid cancer. To develop more targeted individualized prevention and treatment strategies, further analysis from age and sex perspectives is necessary. The 2021 data show that globally, the 20–24 age group bears a substantial thyroid cancer burden, accounting for over 50% of the total prevalence, incidence, and DALYs rates across all age groups. Studies suggest that stress may disrupt thyroid hormone regulation by interfering with the hypothalamic-pituitary-adrenal and hypothalamic-pituitary-thyroid (HPT) axes, indirectly affecting tumor growth (20). Therefore, the high disease burden in this age group may be related to biological, genetic, psychological, and socioenvironmental factors, particularly increased stress, mental health issues, and hormonal instability. Globally and across most regions, the prevalence and incidence rates of thyroid cancer among female children and adolescents exceed those observed in males. This phenomenon may be associated with estrogen levels, which play an important role in thyroid cancer cell proliferation through binding to nuclear receptors estrogen receptor α (ERα) and estrogen receptor β (ERβ), as well as by activating various signaling pathways (e.g., AKT/mTOR, MEK1/2, and MAPK) to stimulate thyroid cancer cell growth (21–23). However, a study examining pediatric thyroid cancer and sex hormone receptors revealed that the female predominance predominantly emerges post-puberty, with no statistically significant differences in the expression of sex hormone receptors identified among younger children. Therefore, the precise causal relationship between gender and pediatric thyroid cancer has yet to be established (24). Obesity, as one of the risk factors for thyroid cancer, contributes to a higher proportion of cancer burden in females (13.1%) than in males (11.9%) globally, including thyroid cancer (25, 26). Therefore, obesity may be a potential explanation for sex differences in pediatric and adolescent thyroid cancer. To identify novel therapeutic targets and offer more precise clinical guidance, further research is warranted to explore the mechanistic role of sex hormone receptors in pediatric thyroid cancer.

The frontier analysis revealed an upward trend in DALYs rates in some high-SDI regions, suggesting potential additional detrimental factors affecting health development. Concurrently, we observed low-SDI countries demonstrating declining DALYs rates despite limited technological and medical resources. This underscores the need for deeper investigation into the drivers of elevated disease burden in high-SDI regions while highlighting these countries’ urgent requirement for more effective preventive measures and treatment protocols. Inequality analysis demonstrated progressively widening disparities between SDI and pediatric/adolescent thyroid cancer DALYs rates over time. By 2021, most high-SDI regions maintained lower DALY rates, with these inequalities showing both persistent and expanding characteristics. These findings indicate that optimized reallocation of medical resources may represent an effective strategy for addressing disease burden disparities.

Pediatric and adolescent thyroid cancer is relatively rare in clinical practice, with particularly low incidence in children under 10 years old (27, 28). However, the incidence increases progressively with age (29). Compared to adults, pediatric thyroid cancer is often more extensive at diagnosis, typically presenting at advanced stages with highly metastatic features (5). Notwithstanding these challenges, the overall prognosis for pediatric thyroid cancer remains relatively favorable. From among 556 patient cases from a study on surgical outcomes for pediatric and adolescent thyroid cancer, there were only 27 (4.9%) cases of early postoperative thyroid nerve injury, and only 7.3% of hypoparathyroidism resulted from thyroid-only surgery. These findings confirm the high safety profile of thyroid cancer surgery, characterized by minimal complications and extended postoperative survival, establishing surgical intervention as an effective therapeutic approach for pediatric and adolescent thyroid cancer (30). Beyond surgery, comprehensive management incorporates endocrine therapy, radiotherapy, and notably, targeted therapies - the latter representing a significant research breakthrough in recent years. Critical evidence demonstrates that larotrectinib, a highly selective tropomyosin receptor kinase (TRK) inhibitor, achieves rapid tumor control in TRK fusion-positive thyroid cancer patients across both adult and pediatric populations, exhibiting remarkable antitumor efficacy (31). In summary, while the incidence, prevalence and DALYs rates of pediatric thyroid cancer continue to rise, the disease generally has a favorable prognosis with relatively low surgical risks and complications. Beyond surgery, endocrine and targeted therapies also play significant roles. Therefore, further research into treatment optimization and disease burden reduction represents an important future direction.

## 5 Limitations

This study has certain limitations. First, the data utilized in our study were sourced from the GBD database, wherein the absence of original data for certain countries resulted in model-estimated figures, potentially leading to an incomplete inclusion of all pediatric and adolescent thyroid cancer cases. Secondly, given the relatively low mortality rate of pediatric and adolescent thyroid cancer, mortality alone is insufficient to fully capture the actual health impact of this disease on this vulnerable population. Therefore, we employed the DALYs metric, which integrates Years of Life Lost due to premature mortality (YLL) and Years Lived with Disability (YLD). This comprehensive measure allows for a more holistic assessment of the disease’s impact on patients’ health-related quality of life and overall healthy lifespan. Analysis of DALYs can identify regions with a high disease burden despite low mortality rates, thereby facilitating targeted research and interventions to reduce the overall burden of disease. Future studies could further explore the mortality aspects of this disease to provide a more comprehensive epidemiological profile of pediatric and adolescent thyroid cancer. Thirdly, the study did not classify pediatric and adolescent thyroid cancer by specific pathological types, while different pathological types may show significant differences in disease characteristics, treatment response and prognosis. Pathological diagnoses and genetic malformations exert significant influence on disease prevalence, incidence, and DALYs rates. Therefore, future research can leverage pathological diagnoses to analyze the burden of different pathological types of diseases, thereby providing enhanced value to the field. Additionally, focusing on pediatric and adolescent thyroid cancer of a specific pathological type may enhance the precision and depth of research. Furthermore, it is recommended to integrate more real-world data into subsequent studies to enhance the accuracy and reliability of research findings through multi-source data validation, thereby offering more valuable references for clinical practice.

## 6 Conclusions

In summary, over the past 32 years, the global burden of pediatric and adolescent thyroid cancer has continued to increase. The disease burden trends vary significantly across regions: most high-income areas have seen gradual reductions in burden due to technological advancements and optimized healthcare resource allocation, while low-income regions have experienced more pronounced increases, likely attributable to population growth, inadequate medical resources, and uneven technological development. Regarding age and sex disparities, the 20–24 age group bears the most severe thyroid cancer burden globally, with females being disproportionately affected compared to males. These findings highlight the pressing necessity for targeted interventions in high-burden regions and nations to alleviate the global disease burden. Rational allocation of medical resources may serve as a pivotal strategy to address this issue. Furthermore, a comprehensive exploration of diverse therapeutic modalities, encompassing surgical interventions, endocrine therapy, and targeted treatments as precision medicine approaches, constitutes a pivotal research direction. This collaborative effort aims to provide patients with optimized therapeutic strategies, thereby improving clinical outcomes and enhancing quality of life.

## Data Availability

The datasets presented in this study can be found in online repositories. The names of the repository/repositories and accession number(s) can be found below: https://ghdx.healthdata.org/gbd-2021/sources.

## References

[1] Boucai L Zafereo M Cabanillas ME. Thyroid cancer: A review. JAMA. (2024) 331:425. doi: 10.1001/jama.2023.26348, PMID: 38319329

[2] Christofer Juhlin C Mete O Baloch ZW. The 2022 WHO classification of thyroid tumors: novel concepts in nomenclature and grading. Endocrine-Related Cancer. (2023) 30:e220293. doi: 10.1530/ERC-22-0293, PMID: 36445235

[3] Pizzato M Li M Vignat J Laversanne M Singh D La Vecchia C. The epidemiological landscape of thyroid cancer worldwide: GLOBOCAN estimates for incidence and mortality rates in 2020. Lancet Diabetes Endocrinol. (2022) 10:264–72. doi: 10.1016/S2213-8587(22)00035-3, PMID: 35271818

[4] Kitahara CM Schneider AB. Epidemiology of thyroid cancer. Cancer Epidemiology Biomarkers Prev. (2022) 31:1284–97. doi: 10.1158/1055-9965.EPI-21-1440, PMID: 35775227 PMC9473679

[5] Lamartina L Leboulleux S Schlumberger M. Thyroid cancer incidence in children and adolescents. Lancet Diabetes Endocrinol. (2021) 9:128–9. doi: 10.1016/S2213-8587(20)30430-7, PMID: 33482108

[6] Thiesmeyer JW Egan CE Greenberg JA Beninato T Zarnegar R Fahey Iii TJ. Prepubertal children with papillary thyroid carcinoma present with more invasive disease than adolescents and young adults. Thyroid. (2023) 33:214–22. doi: 10.1089/thy.2022.0098, PMID: 36355601

[7] Siegel RL Miller KD Wagle NS Jemal A. Cancer statistics, 2023. CA Cancer J Clin. (2023) 73:17–48. doi: 10.3322/caac.21763, PMID: 36633525

[8] Ferrari AJ Santomauro DF Aali A Abate YH Abbafati C Abbastabar H.Global incidence, prevalence, years lived with disability (YLDs), disability-adjusted life-years (DALYs), and healthy life expectancy (HALE) for 371 diseases and injuries in 204 countries and territories and 811 subnational locations, 1990-2021: a systematic analysis for the Global Burden of Disease Study 2021. Lancet. (2024) 403:2133–61. doi: 10.1016/S0140-6736(24)00757-8, PMID: 38642570 PMC11122111

[9] Mokdad AH Bisignano C Hsu JM Ababneh HS Abbasgholizadeh R Abdelkader A. The burden of diseases, injuries, and risk factors by state in the USA, 1990-2021: a systematic analysis for the Global Burden of Disease Study 2021. Lancet. (2024) 404:2314–40. doi: 10.1016/S0140-6736(24)01446-6, PMID: 39645376 PMC11694014

[10] Cheng F Xiao J Shao C Huang F Wang L Ju Y. Burden of thyroid cancer from 1990 to 2019 and projections of incidence and mortality until 2039 in China: findings from global burden of disease study. Front Endocrinol. (2021) 12:738213. doi: 10.3389/fendo.2021.738213, PMID: 34690931 PMC8527095

[11] You L Lv Z Li C Ye W Zhou Y Jin J. Worldwide cancer statistics of adolescents and young adults in 2019: a systematic analysis of the Global Burden of Disease Study 2019. ESMO Open. (2021) 6:100255. doi: 10.1016/j.esmoop.2021.100255, PMID: 34481330 PMC8417345

[12] Murray CJL Abbafati C Abbas KM Abbasi M Abbasi-Kangevari M Abd-Allah F.Five insights from the global burden of disease study 2019. Lancet. (2020) 396:1135–59. doi: 10.1016/S0140-6736(20)31404-5, PMID: 33069324 PMC7116361

[13] Bai Z Wang H Shen C An J Yang Z Mo X. The global, regional, and national patterns of change in the burden of non-malignant upper gastrointestinal diseases from 1990 to 2019 and the forecast for the next decade. Int J Surg. (2024) 111:80–92. doi: 10.1097/JS9.0000000000001902, PMID: 38959095 PMC11745775

[14] Ward JL Azzopardi PS Francis KL Santelli JS Skirbekk V Sawyer SM.Global, regional, and national mortality among young people aged 10–24 years, 1950-2019: a systematic analysis for the Global Burden of Disease Study 2019. Lancet. (2021) 398:1593–618. doi: 10.1016/S0140-6736(21)01546-4, PMID: 34755628 PMC8576274

[15] Yang X Zhang T Zhang X Chu C Sang S. Global burden of lung cancer attributable to ambient fine particulate matter pollution in 204 countries and territories, 1990-2019. Environ Res. (2022) 204:112023. doi: 10.1016/j.envres.2021.112023, PMID: 34520750

[16] Ding Q Liu S Yao Y Liu H Cai T Han L. Global, regional, and national burden of ischemic stroke, 1990-2019. Neurology. (2022) 98:e279–90. doi: 10.1212/WNL.0000000000013115, PMID: 34911748

[17] Alzahrani AS Alkhafaji D Tuli M Al-Hindi H Sadiq BB. Comparison of differentiated thyroid cancer in children and adolescents (≤20 years) with young adults. Clin Endocrinol. (2016) 84:571–7. doi: 10.1111/cen.12845, PMID: 26118454

[18] Trama A Stark D Bozovic-Spasojevic I Gaspar N Peccatori F Toss A. Cancer burden in adolescents and young adults in Europe. ESMO Open. (2023) 8:100744. doi: 10.1016/j.esmoop.2022.100744, PMID: 36753992 PMC10024081

[19] Drozd V Saenko V Branovan DI Brown K Yamashita S Reiners C. A search for causes of rising incidence of differentiated thyroid cancer in children and adolescents after chernobyl and fukushima: comparison of the clinical features and their relevance for treatment and prognosis. Int J Environ Res Public Health. (2021) 18(7):3444. doi: 10.3390/ijerph18073444, PMID: 33810323 PMC8037740

[20] Kyriacou A Tziaferi V Toumba M. Stress, thyroid dysregulation, and thyroid cancer in children and adolescents: proposed impending mechanisms. Horm Res Paediatr. (2023) 96:44–53. doi: 10.1159/000524477, PMID: 35385843

[21] Halada S Casado-Medrano V Baran JA Lee J Chinmay P Bauer AJ. Hormonal crosstalk between thyroid and breast cancer. Endocrinology. (2022) 163(7):bqac075. doi: 10.1210/endocr/bqac075, PMID: 35587175 PMC9653009

[22] Liu J Xu T Ma L Chang W. Signal pathway of estrogen and estrogen receptor in the development of thyroid cancer. Front Oncol. (2021) 11:593479. doi: 10.3389/fonc.2021.593479, PMID: 33996538 PMC8113849

[23] Gong Z Yang S Wei M Vlantis AC Chan JYK van Hasselt CA. The isoforms of estrogen receptor alpha and beta in thyroid cancer. Front Oncol. (2022) 12:916804. doi: 10.3389/fonc.2022.916804, PMID: 35814443 PMC9263191

[24] da Silva Breder JRA Alves PAG Araújo ML Pires B Valverde P Bulzico DA. Puberty and sex in pediatric thyroid cancer: could expression of estrogen and progesterone receptors affect prognosis? Eur Thyroid J. (2022) 11:e210090. doi: 10.1530/ETJ-21-0090, PMID: 35113037 PMC8963171

[25] Avgerinos KI Spyrou N Mantzoros CS Dalamaga M. Obesity and cancer risk: Emerging biological mechanisms and perspectives. Metabolism. (2019) 92:121–35. doi: 10.1016/j.metabol.2018.11.001, PMID: 30445141

[26] Franchini F Palatucci G Colao A Ungaro P Macchia PE Nettore IC. Obesity and thyroid cancer risk: an update. Int J Environ Res Public Health. (2022) 19(3):1116. doi: 10.3390/ijerph19031116, PMID: 35162142 PMC8834607

[27] Siegel DA King JB Lupo PJ Durbin EB Tai E Mills K. Counts, incidence rates, and trends of pediatric cancer in the United States, 2003-2019. J Natl Cancer Inst. (2023) 115:1337–54. doi: 10.1093/jnci/djad115, PMID: 37433078 PMC11018256

[28] Liu Y Wang S Li Y Zhang X Liu Z Liu Q. Clinical Heterogeneity of Differentiated Thyroid Cancer between Children Less than 10 Years of Age and Those Older than 10 Years: A Retrospective Study of 70 Cases. Eur Thyroid J. (2021) 10:364–71. doi: 10.1159/000516830, PMID: 34540706 PMC8406248

[29] Vaccarella S Lortet-Tieulent J Colombet M Davies L Stiller CA Schüz J. Global patterns and trends in incidence and mortality of thyroid cancer in children and adolescents: a population-based study. Lancet Diabetes Endocrinol. (2021) 9:144–52. doi: 10.1016/S2213-8587(20)30401-0, PMID: 33482107

[30] Weber T Hummel R Vorländer C Zielke A Hermann M Krappitz A. Thyroid surgery in children and adolescents: results from a multi-institutional German and Austrian database. Br J Surg. (2023) 110:1808–14. doi: 10.1093/bjs/znad255, PMID: 37758484

[31] Waguespack SG Drilon A Lin JJ Brose MS McDermott R Almubarak M. Efficacy and safety of larotrectinib in patients with TRK fusion-positive thyroid carcinoma. Eur J Endocrinol. (2022) 186:631–43. doi: 10.1530/EJE-21-1259, PMID: 35333737 PMC9066591

