# Supplementary material for: Global, regional, and national burden of Pediatric and adolescent thyroid cancer from 1990 to 2021: a statistical analysis of prevalence, incidence, and DALYs
Source: Front Oncol. 2025 Jul 29;15:1630648. doi: 10.3389/fonc.2025.1630648 (PMC12340228; doi:10.3389/fonc.2025.1630648)
Supplement: Supplementary file 1 [file DataSheet1.zip › Additional files/Additional file1(FigureS1-S12)/Additional FigureS1-S12.pdf]

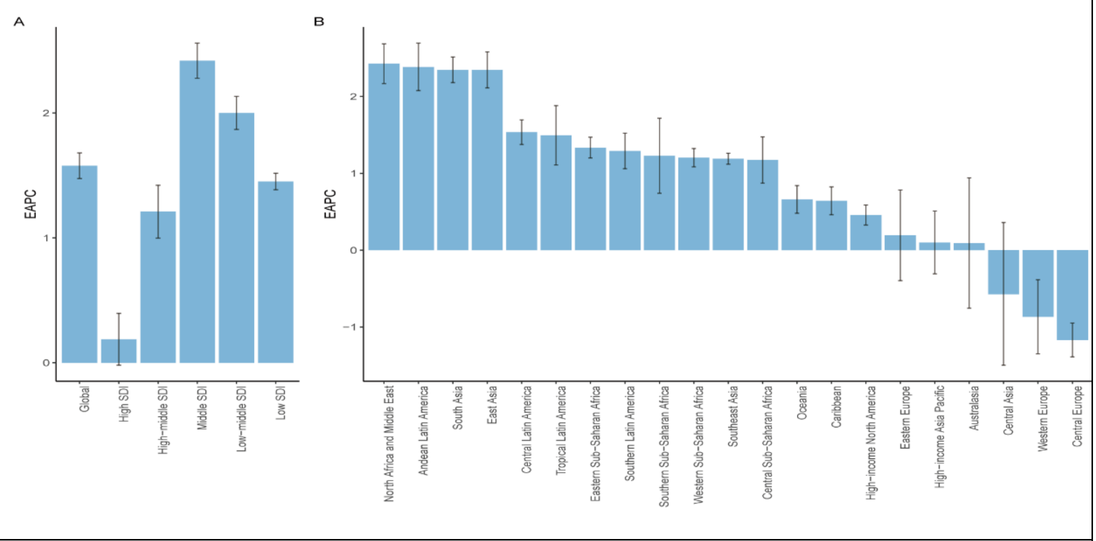

Figure S1. The EAPC of Prevalence Rates for Thyroid Cancer in Children and Adolescents From 1990 to 2021. A Global and SDI regional. B 21 Geographic regions.

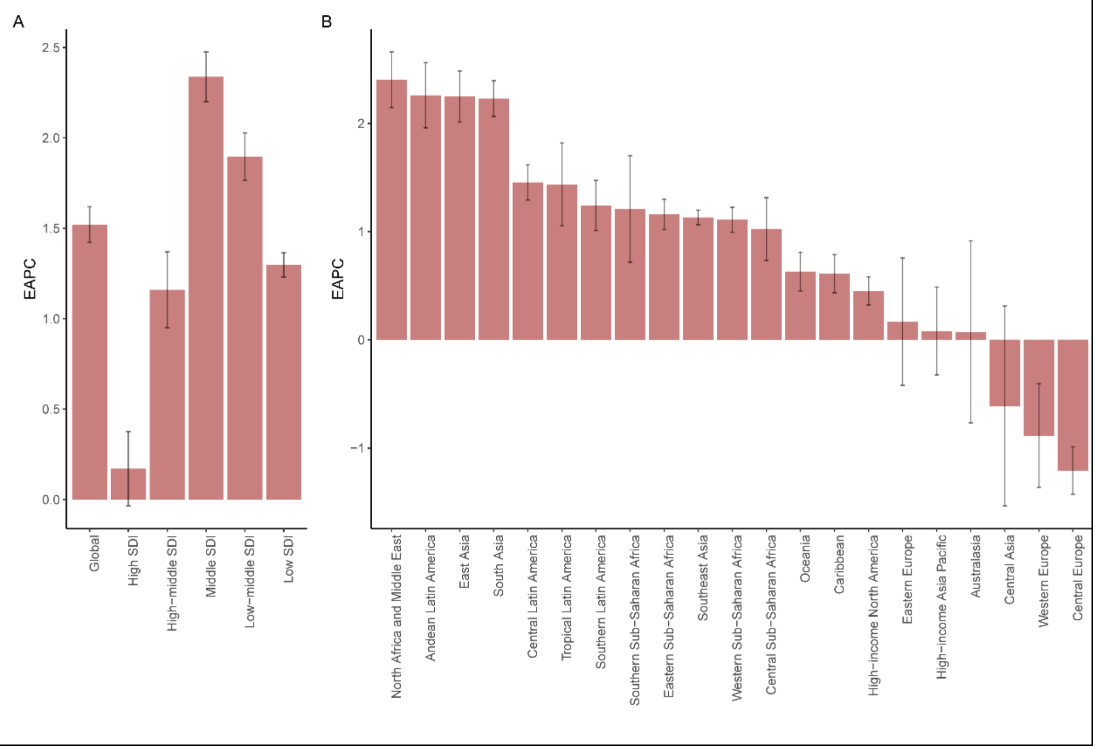

Figure S2. The EAPC of Incidence Rates for Thyroid Cancer in Children and Adolescents From 1990 to 2021. A Global and SDI regional. B 21 Geographic regions.

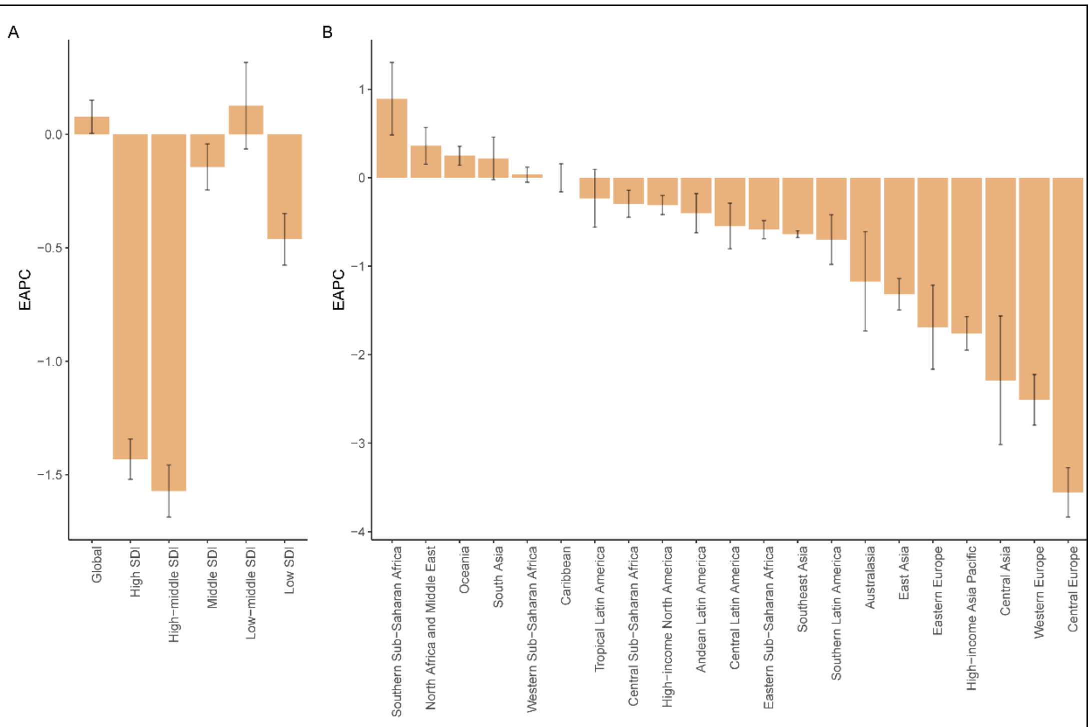

Figure S3. The EAPC of Disability-Adjusted Life-Years (DALYs) Rates for Thyroid Cancer in Children and Adolescents From 1990 to 2021. A Global and SDI regional. B 21 Geographic regions.

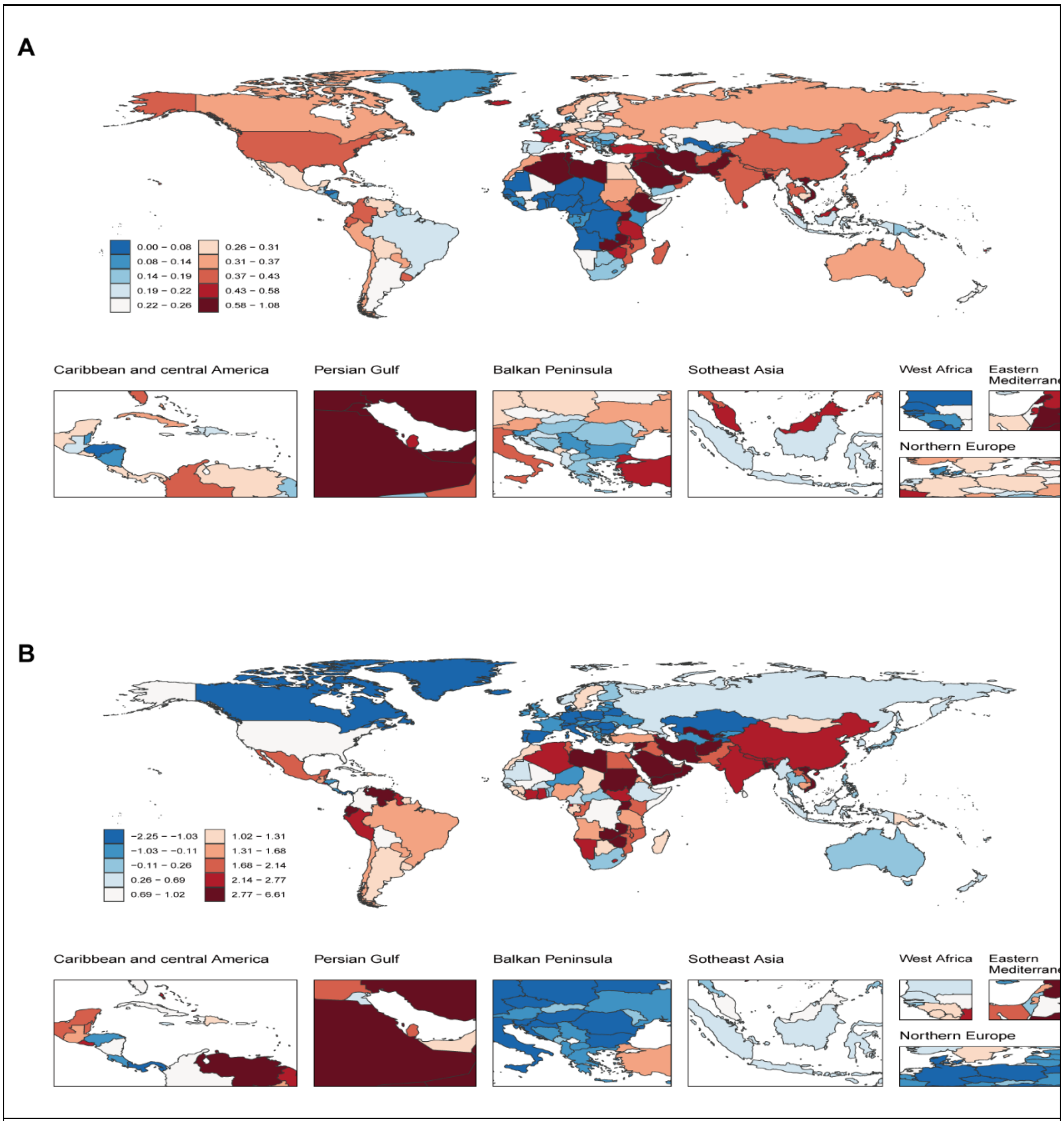

Figure S4. Temporal trend of thyroid cancer in children and adolescents globally. A Percentage change in incident cases across 204 countries in 1990 and 2021. B EAPC in incidence rates across 204 countries from 1990 to 2021. EAPC = Estimated Annual Percentage Change

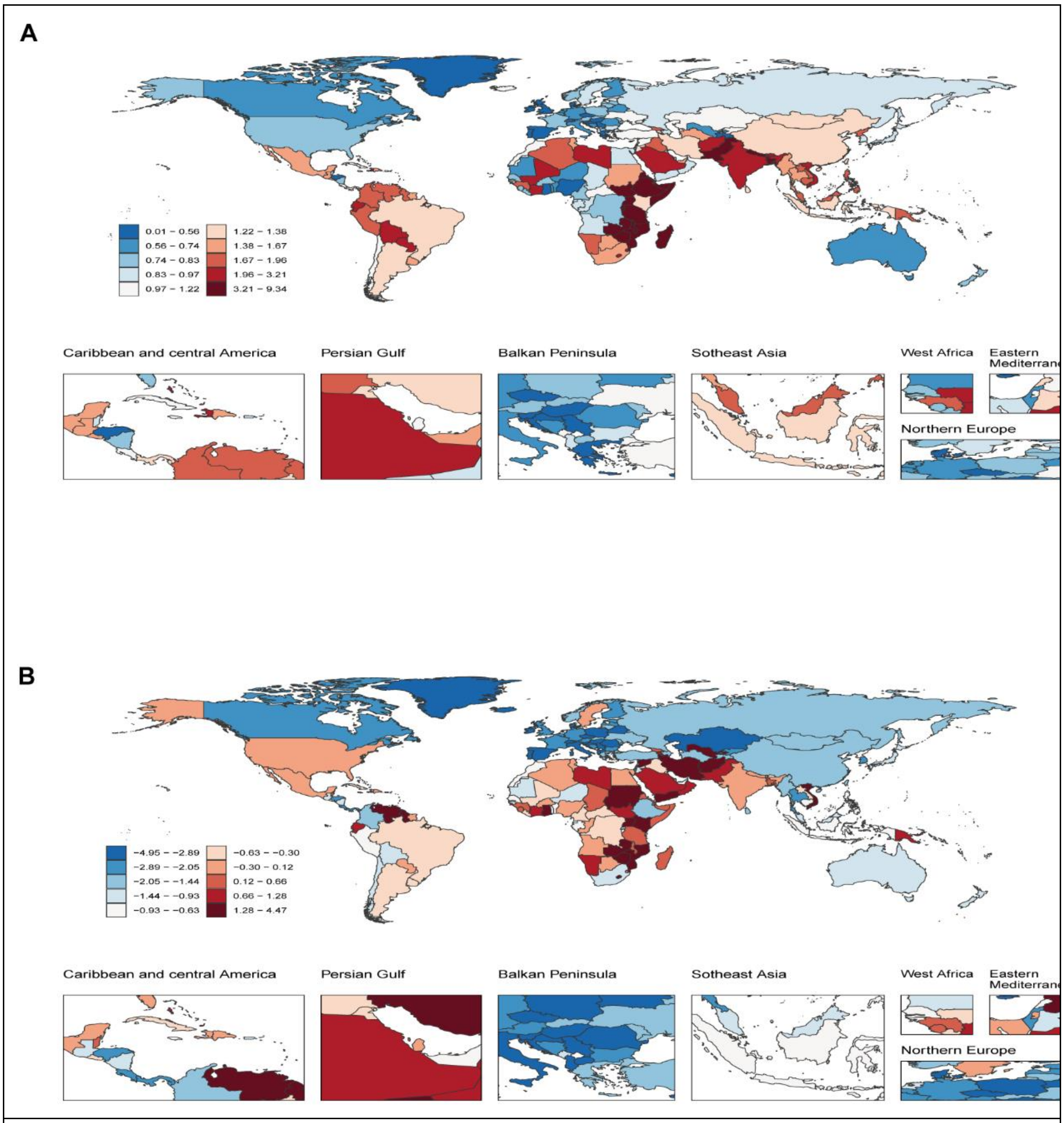

Figure S5. Temporal trend of thyroid cancer in children and adolescents globally. A Percentage change in DALYs cases across 204 countries in 1990 and 2021. B EAPC in DALYs rates across 204 countries from 1990 to 2021. EAPC = Estimated Annual Percentage Change

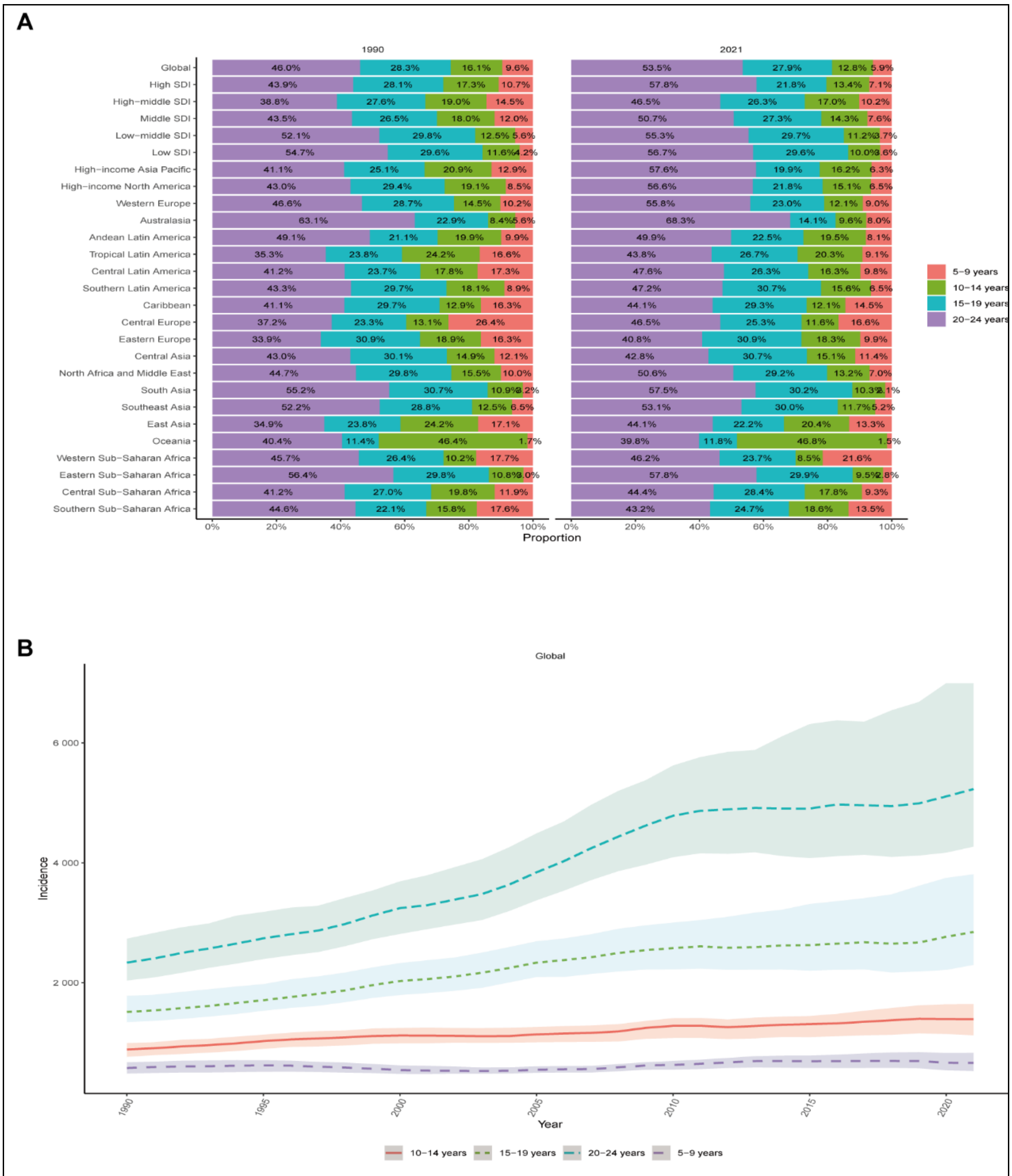

Figure S6. Temporal trend of Thyroid Cancer in Children and Adolescents by age pattern at the Global Level. A: The distribution of incidence rates across 4 age groups as percentages globally, in 5 territories, and 21 GBD regions in 1990 and 2021. B: Trends of incidence Rates in 4 age groups of Thyroid Cancer in Children and Adolescents From 1990 to 2021.

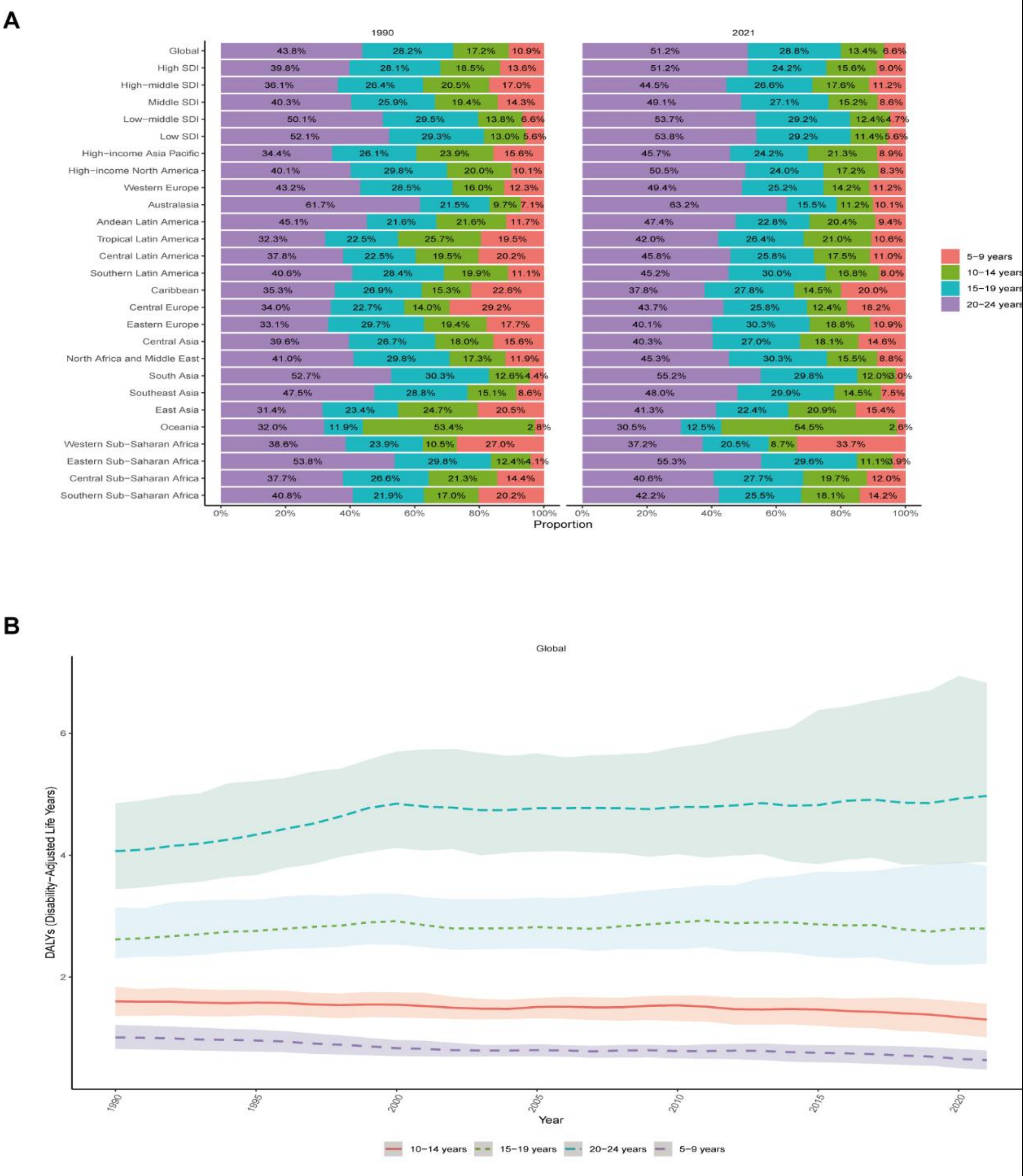

Figure S7. Temporal trend of Thyroid Cancer in Children and Adolescents by age pattern at the Global Level. A: The distribution of DALYs rates across 4 age groups as percentages globally, in 5 territories, and 21 GBD regions in 1990 and 2021. B: Trends of DALYs Rates in 4 age groups of Thyroid Cancer in Children and Adolescents From 1990 to 2021.

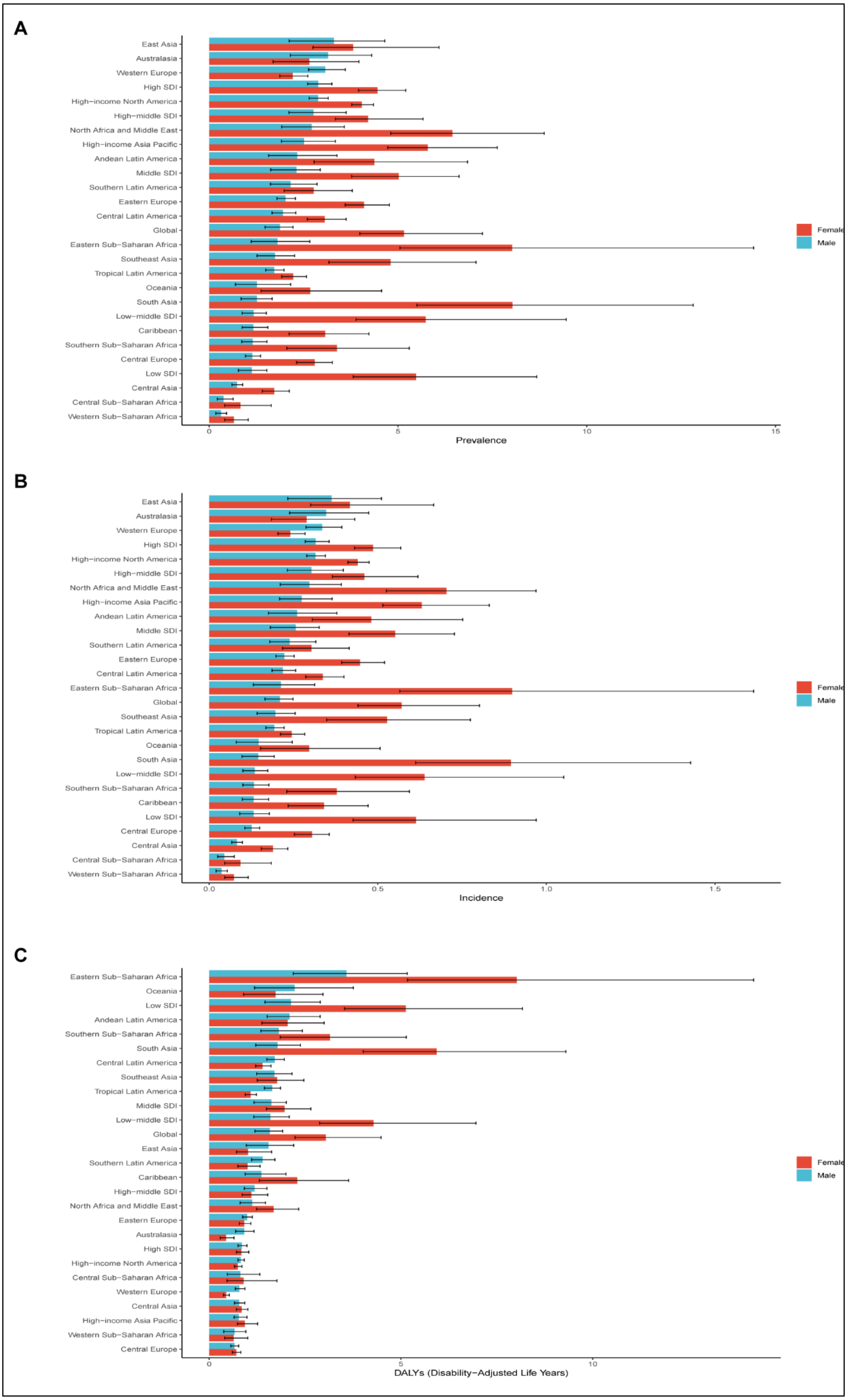

A
East Asia
Australasia
Western Europe
High SDI
High−income North America
High−middle SDI
North Africa and Middle East
High−income Asia Pacific
Andean Latin America
Middle SDI
Southern Latin America
Eastern Europe
Central Latin America
Global
Eastern Sub−Saharan Africa
Southeast Asia
Tropical Latin America
Oceania
South Asia
Low−middle SDI
Caribbean
Southern Sub−Saharan Africa
Central Europe
Low SDI
Central Asia
Central Sub−Saharan Africa
Western Sub−Saharan Africa
0
5
10
15
Prevalence
Female
Male
B
East Asia
Australasia
Western Europe
High SDI
High−income North America
High−middle SDI
North Africa and Middle East
High−income Asia Pacific
Andean Latin America
Middle SDI
Southern Latin America
Eastern Europe
Central Latin America
Eastern Sub−Saharan Africa
Global
Southeast Asia
Tropical Latin America
Oceania
South Asia
Low−middle SDI
Southern Sub−Saharan Africa
Caribbean
Low SDI
Central Europe
Central Asia
Central Sub−Saharan Africa
Western Sub−Saharan Africa
0.0
0.5
1.0
1.5
Incidence
Female
Male
C
Eastern Sub−Saharan Africa
Oceania
Low SDI
Andean Latin America
Southern Sub−Saharan Africa
South Asia
Central Latin America
Southeast Asia
Tropical Latin America
Middle SDI
Low−middle SDI
Global
East Asia
Southern Latin America
Caribbean
High−middle SDI
North Africa and Middle East
Eastern Europe
Australasia
High SDI
High−income North America
Central Sub−Saharan Africa
Western Europe
Central Asia
High−income Asia Pacific
Western Sub−Saharan Africa
Central Europe
0
5
10
DALYs (Disability−Adjusted Life Years)
Female
Male

Figure S8. Sex analysis of thyroid cancer among children and adolescents burden in 2021. A Prevalence rates. B Incidence rates. C DALYs rates.

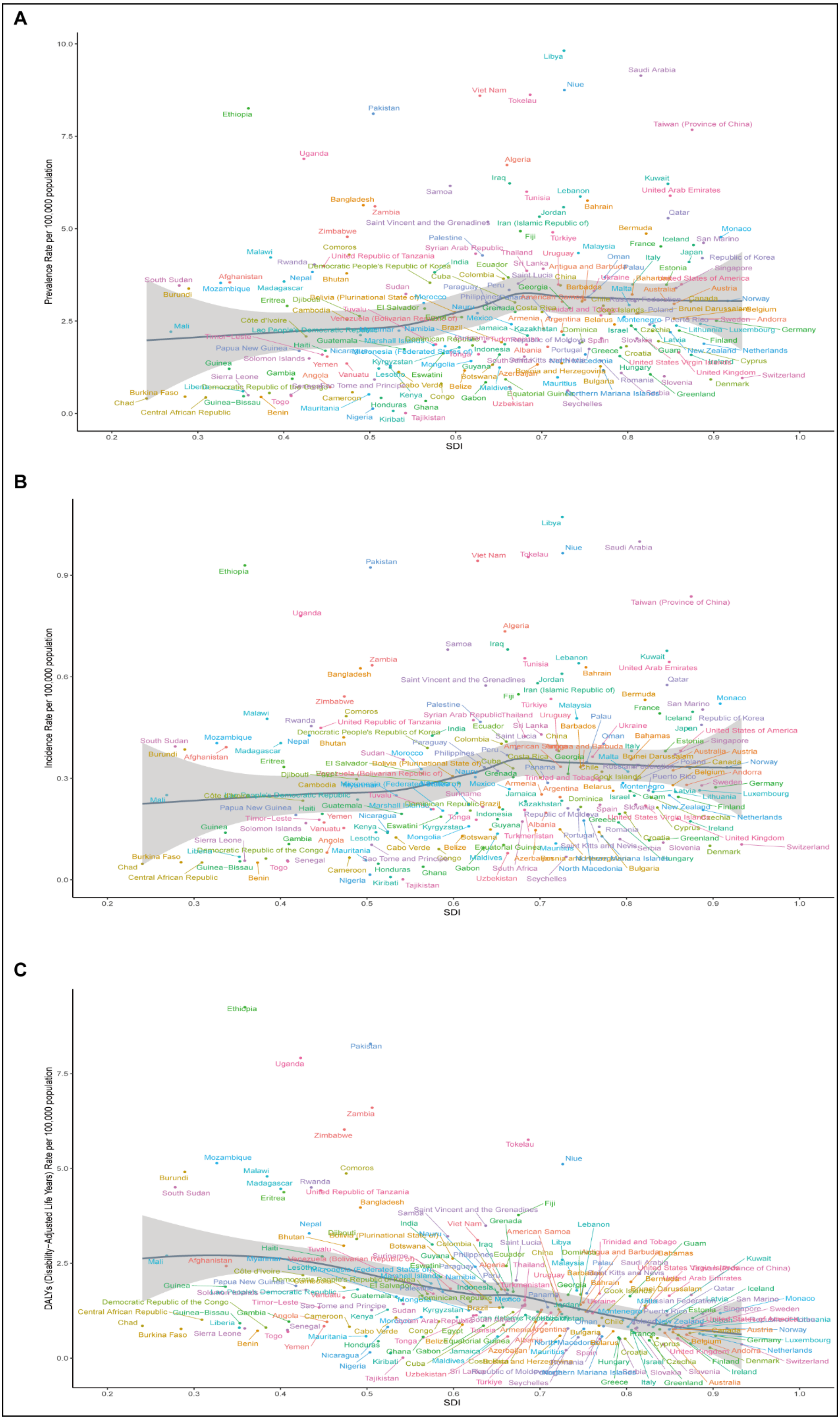
A
Prevalence Rate per 100,000 population
10.0
7.5
5.0
2.5
0.0
0.2
0.3
0.4
0.5
0.6
0.7
0.8
0.9
1.0
SDI
B
Incidence Rate per 100,000 population
0.9
0.6
0.3
0.0
SDI
C
DALYs (Disability−Adjusted Life Years) Rate per 100,000 population
7.5
5.0
2.5
0.0
SDI

Figure S9. The association between the SDI and the prevalence, incidence and DALYS rates of thyroid cancer among children and adolescents across 204 countries in 2021. A The association between the SDI and the prevalence rate. B The association between the SDI and the incidence rate. C The association between the SDI and the DALYS rate. SDI = Socio-Demographic Index.

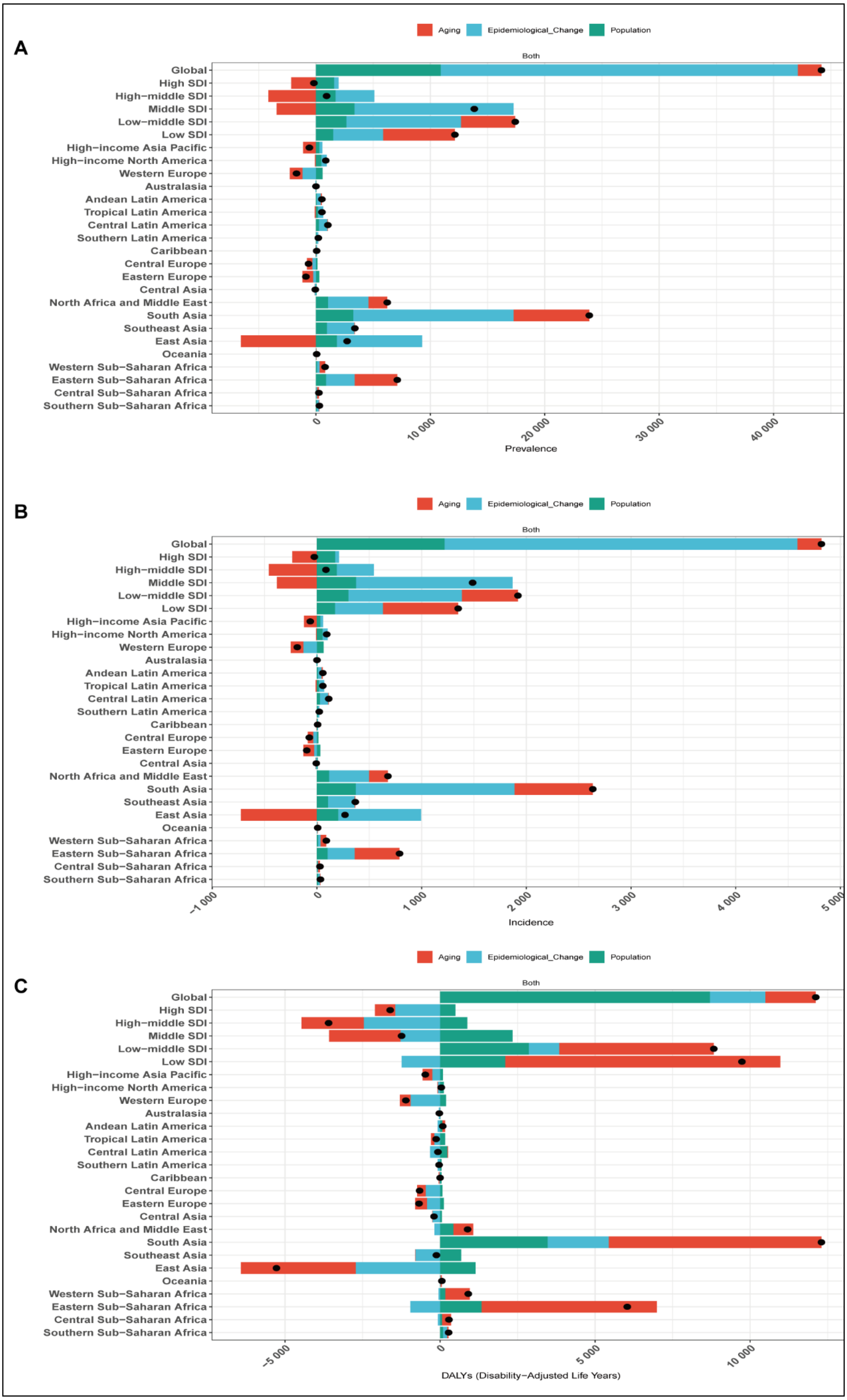
A
Aging
Epidemiological_Change
Population
Both
Global
High SDI
High−middle SDI
Middle SDI
Low−middle SDI
Low SDI
High−income Asia Pacific
High−income North America
Western Europe
Australasia
Andean Latin America
Tropical Latin America
Central Latin America
Southern Latin America
Caribbean
Central Europe
Eastern Europe
Central Asia
North Africa and Middle East
South Asia
Southeast Asia
East Asia
Oceania
Western Sub−Saharan Africa
Eastern Sub−Saharan Africa
Central Sub−Saharan Africa
Southern Sub−Saharan Africa
0
10 000
20 000
30 000
40 000
Prevalence
B
Incidence
−1 000
0
1 000
2 000
3 000
4 000
5 000
C
DALYs (Disability−Adjusted Life Years)
−5 000
0
5 000
10 000

Figure S10. Changes in the prevalence, incidence, and DALYs of thyroid cancer among children and adolescents from 1990 to 2019, according to population-level determinants of population growth, aging, and epidemiological changes at the global level and by SDI quintile. A Changes in the prevalence rate. B Changes in the incidence rate. C Changes in the DALYs rate. The black dot represents the overall value of change contributed by all 3 components. For each component, a positive value indicates an increase in the prevalence, incidence, and DALYs of thyroid cancer among children and adolescents, attributed to the respective component; a negative value indicates a decrease in the prevalence, incidence, and DALYs of thyroid cancer among children and adolescents, attributed to the respective component. SDI: Socio-demographic index; DALYs: Disability-Adjusted Life Years.

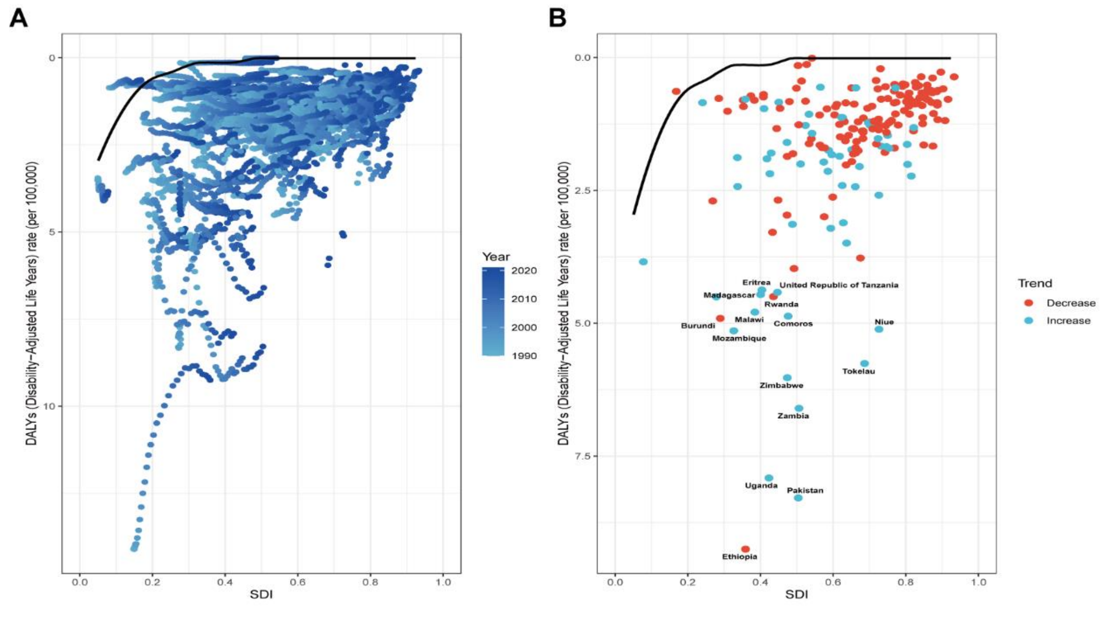

Figure S11. Frontier Analysis of DALYs for Pediatric and Adolescent Thyroid Cancer and SDI in 2021. The color gradient ranges from 1990 (light blue) to 2020 (dark blue). The black curve outlines the frontier, with points representing countries and regions. The top 15 countries with the largest effective disparities are marked in black. Red dots indicate a decrease in the Age-Standardized Disability-Adjusted Life Year Rate (ASDR) for pediatric and adolescent thyroid cancer from 1990 to 2021, while blue dots indicate an increase. ASDR： Age-Standardized Disability-Adjusted Life Year Rate; SDI: Socio-demographic index; DALYs: Disability-Adjusted Life Years

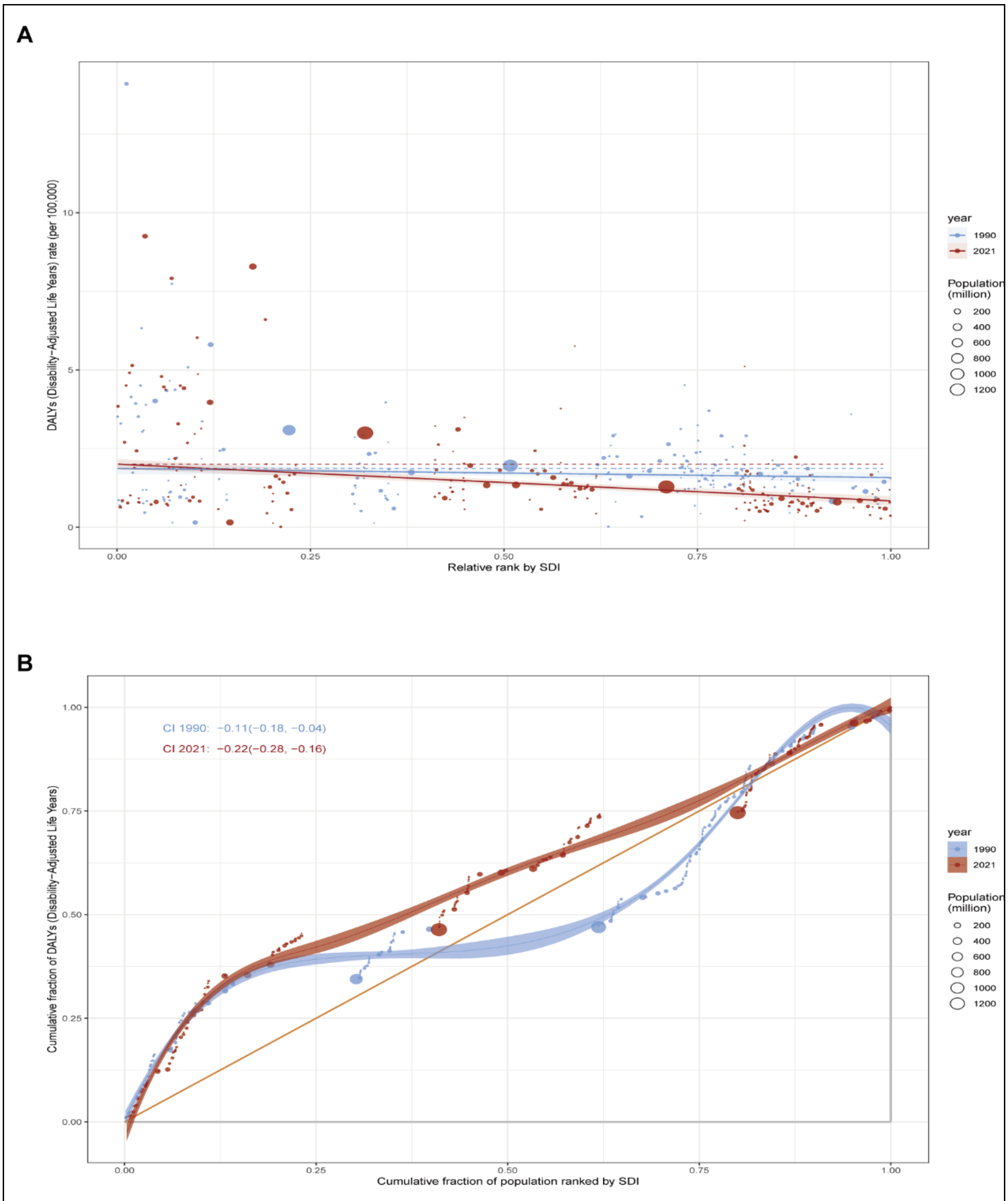

Figure S12. Global Slope Index of Inequality (SII) and Concentration Index (CI) for pediatric and adolescent thyroid cancer DALYs in 1990 and 2021. Circles represent countries with different population sizes. Blue indicates 1990, red indicates 2021. Figure A displays the Slope Index of Inequality, illustrating the relationship between SDI and age-standardized DALYs rates. Figure B present the concentration index, which quantifies relative inequalities by integrating the area under the Lorenz curve, aligning DALYs distribution with population distribution by SDI. SDI: Socio-demographic index; DALYs: Disability-Adjusted Life Years
