# Supplementary figures and images for: Global, regional, and national burden of Pediatric and adolescent thyroid cancer from 1990 to 2021: a statistical analysis of prevalence, incidence, and DALYs
Source: Front Oncol. 2025 Jul 29;15:1630648. doi: 10.3389/fonc.2025.1630648 (PMC12340228; doi:10.3389/fonc.2025.1630648)

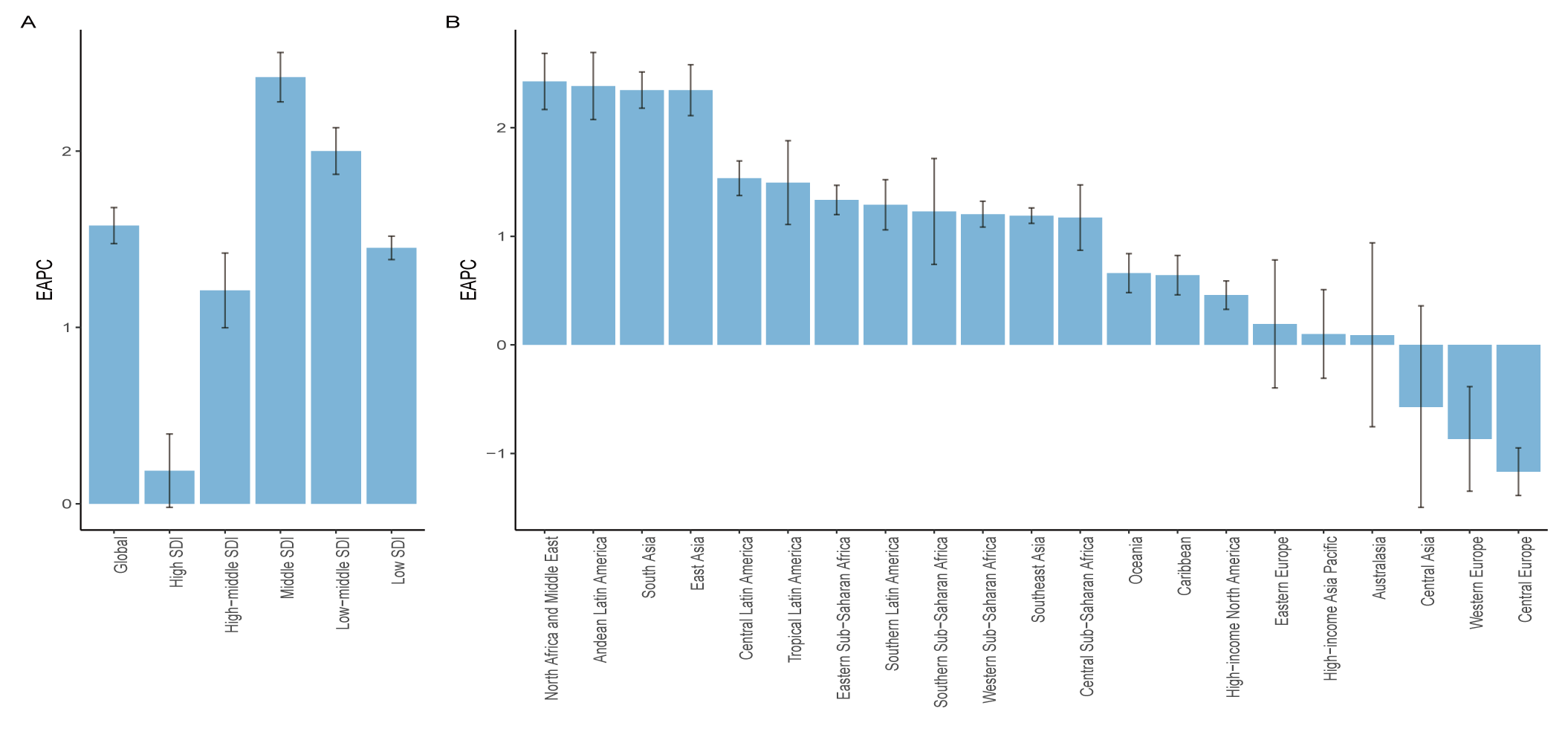

Supplement: Supplementary file 1 [file DataSheet1.zip › Additional files/Additional file1(FigureS1-S12)/FigureS1.tif]

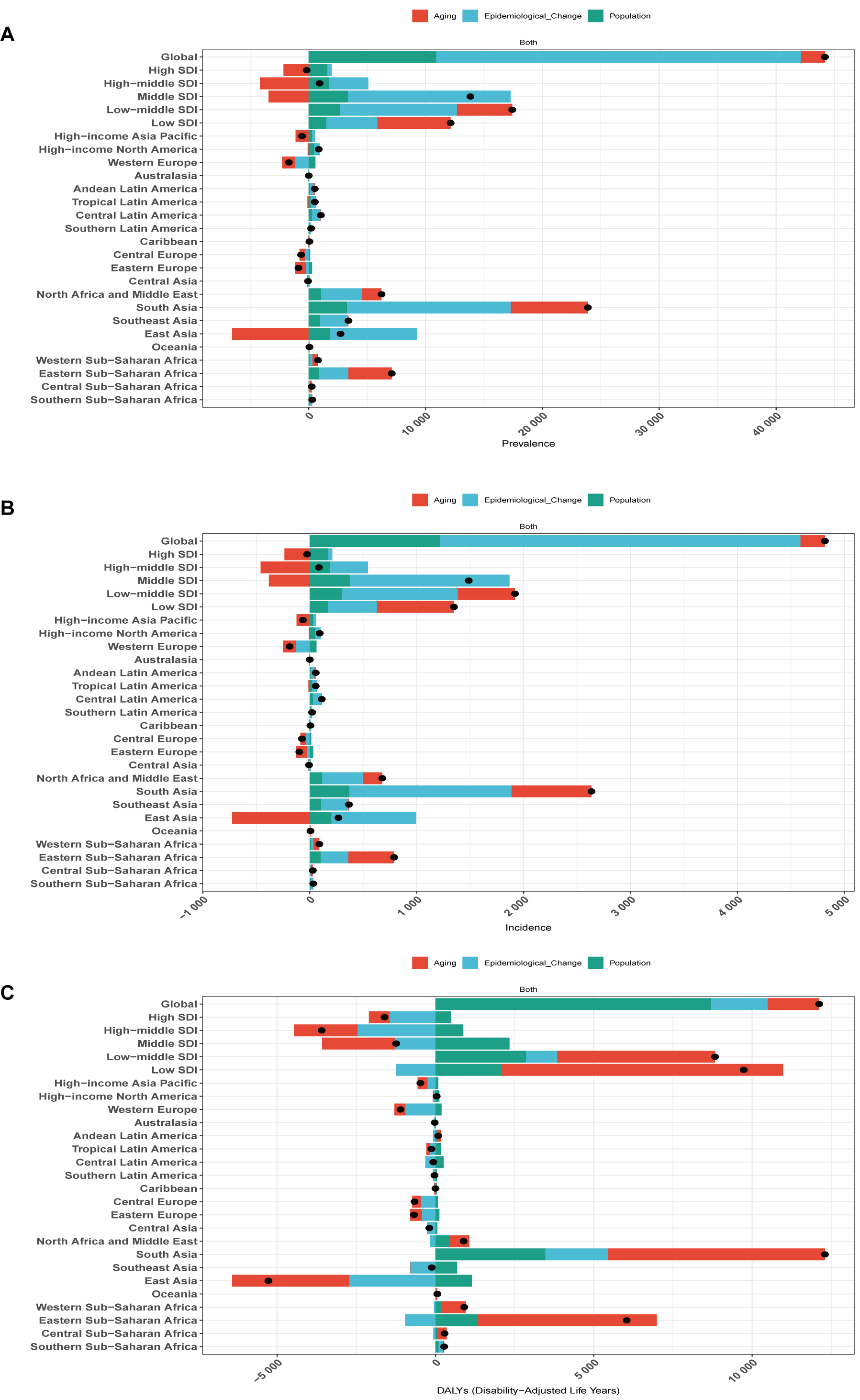

Supplement: Supplementary file 1 [file DataSheet1.zip › Additional files/Additional file1(FigureS1-S12)/FigureS10.tif]

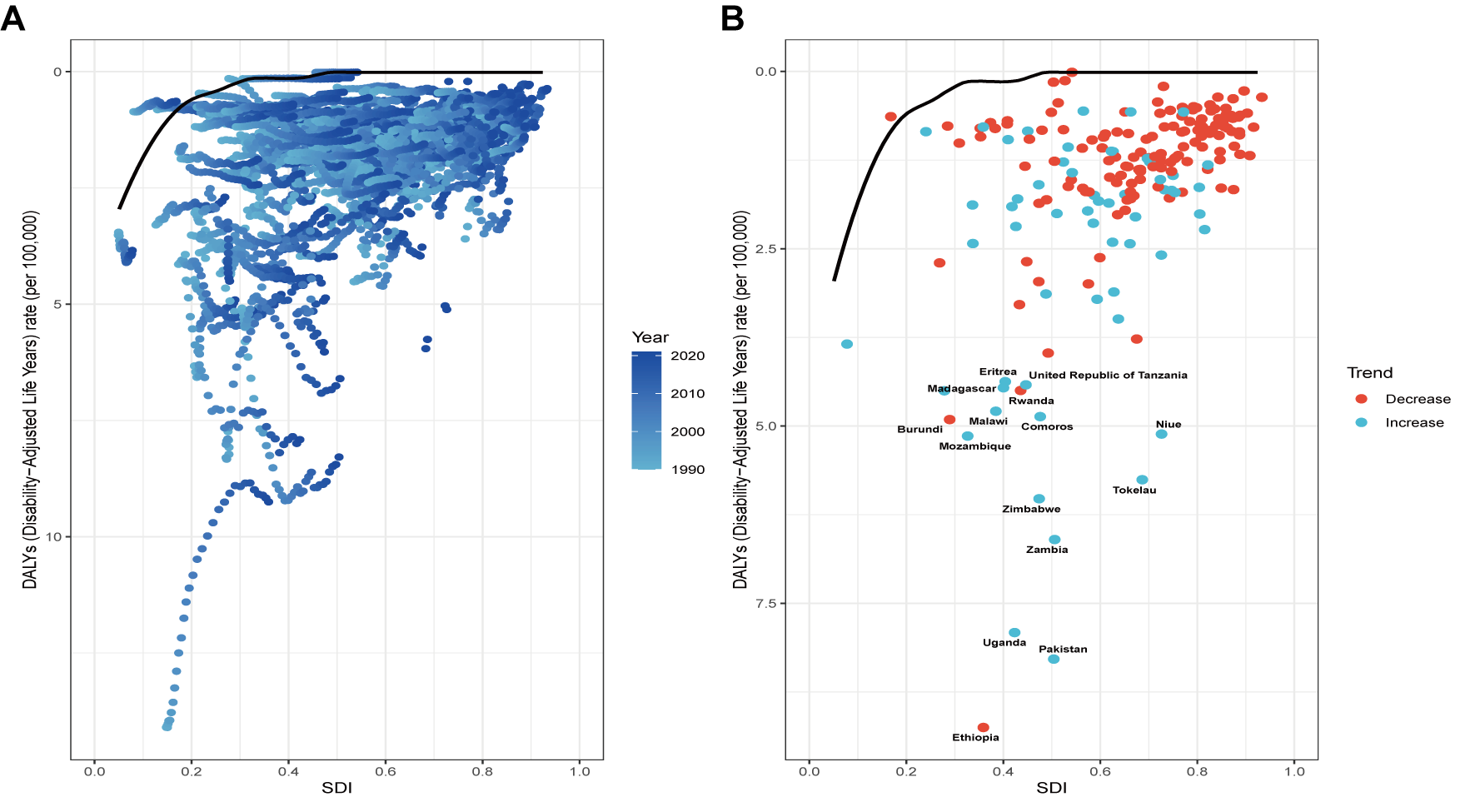

Supplement: Supplementary file 1 [file DataSheet1.zip › Additional files/Additional file1(FigureS1-S12)/FigureS11.tif]

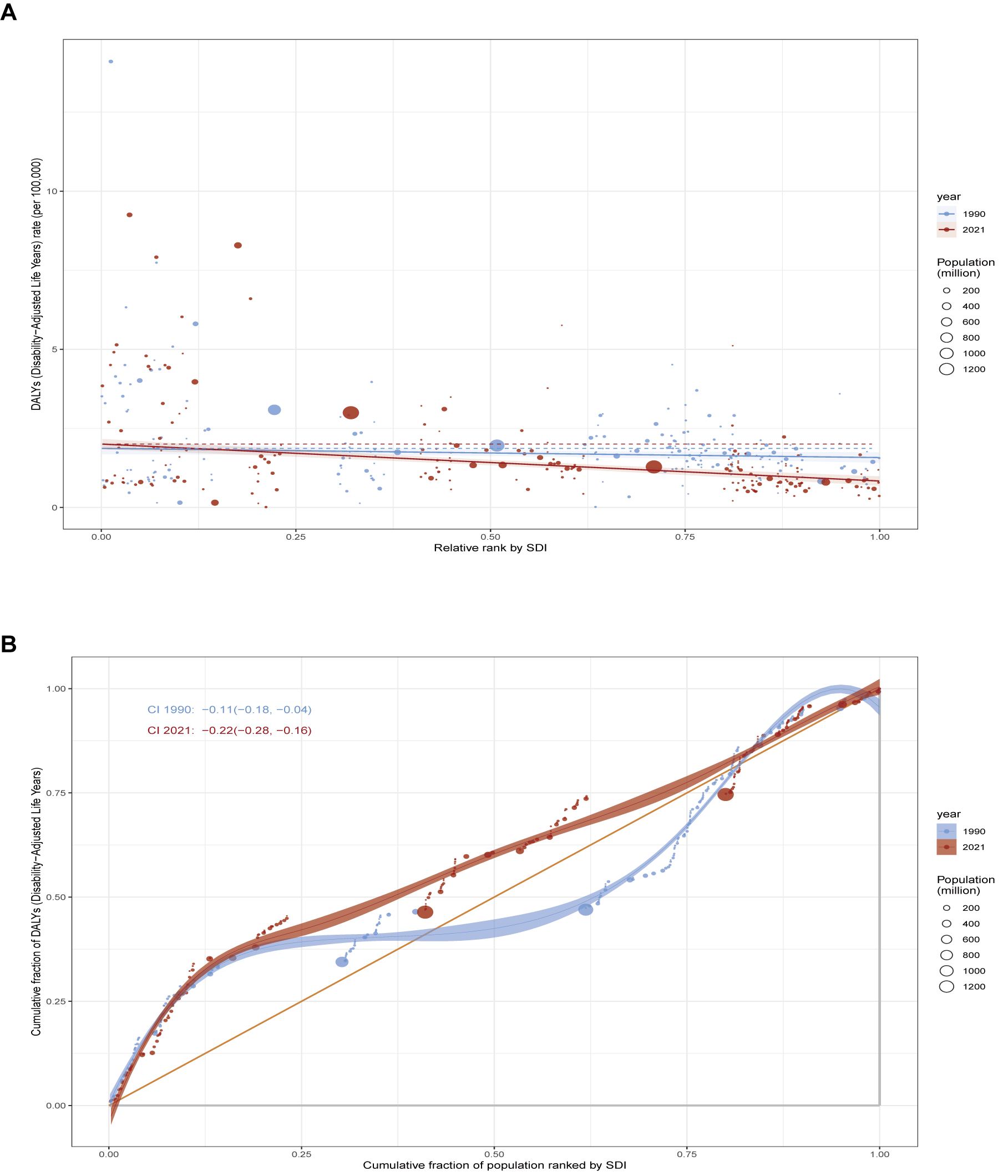

Supplement: Supplementary file 1 [file DataSheet1.zip › Additional files/Additional file1(FigureS1-S12)/FigureS12.tif]

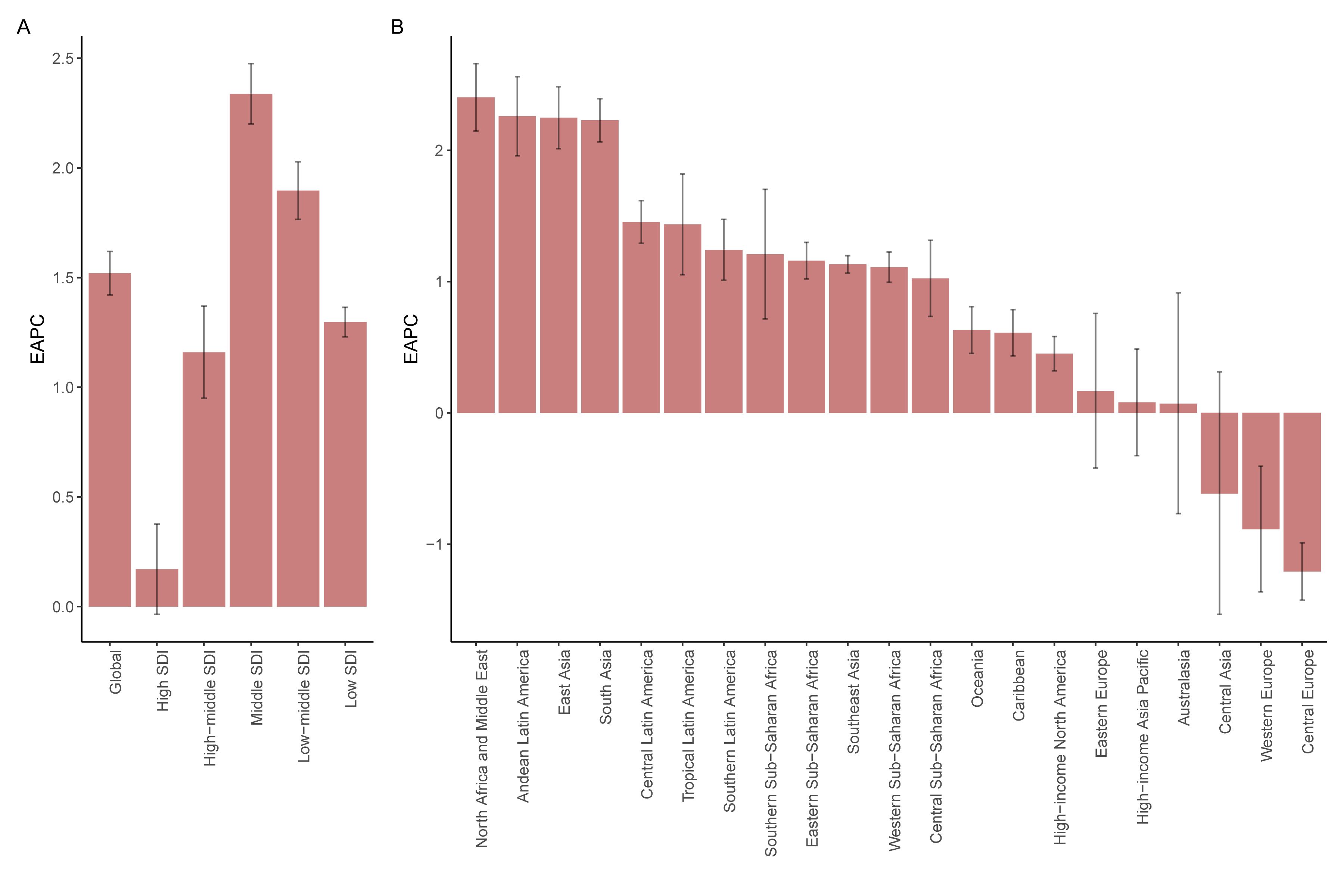

Supplement: Supplementary file 1 [file DataSheet1.zip › Additional files/Additional file1(FigureS1-S12)/FigureS2.tif]

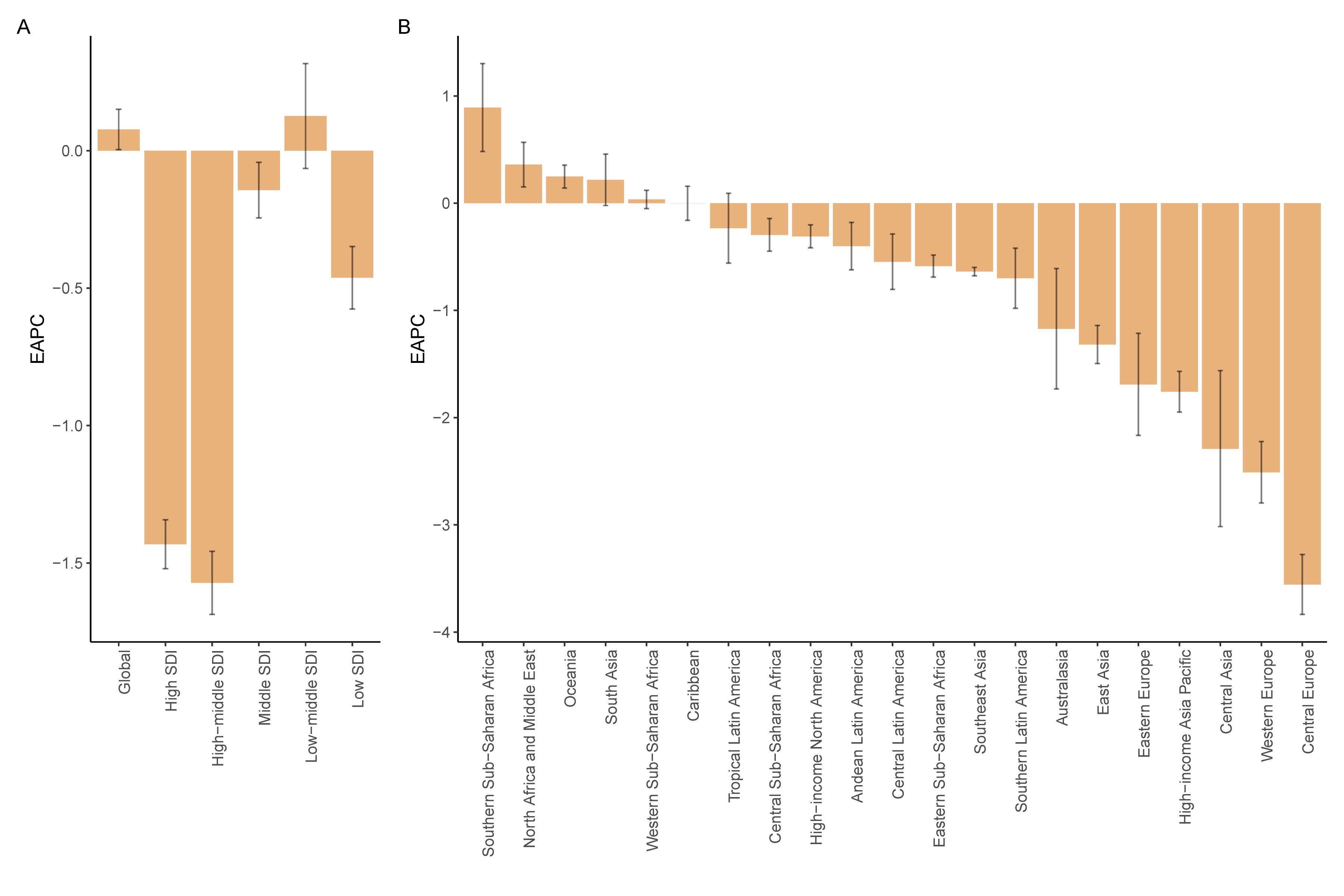

Supplement: Supplementary file 1 [file DataSheet1.zip › Additional files/Additional file1(FigureS1-S12)/FigureS3.tif]

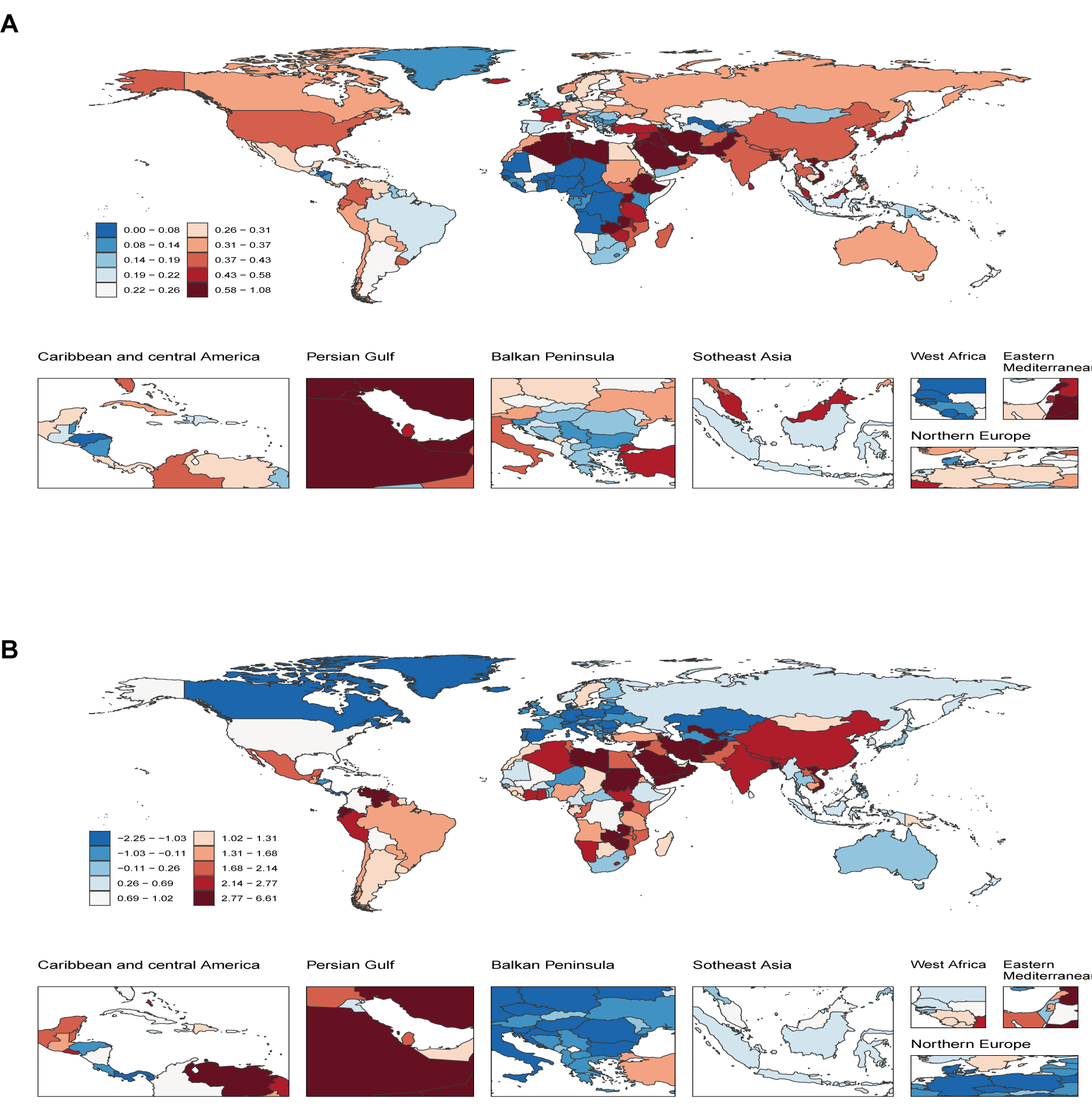

Supplement: Supplementary file 1 [file DataSheet1.zip › Additional files/Additional file1(FigureS1-S12)/FigureS4.tif]

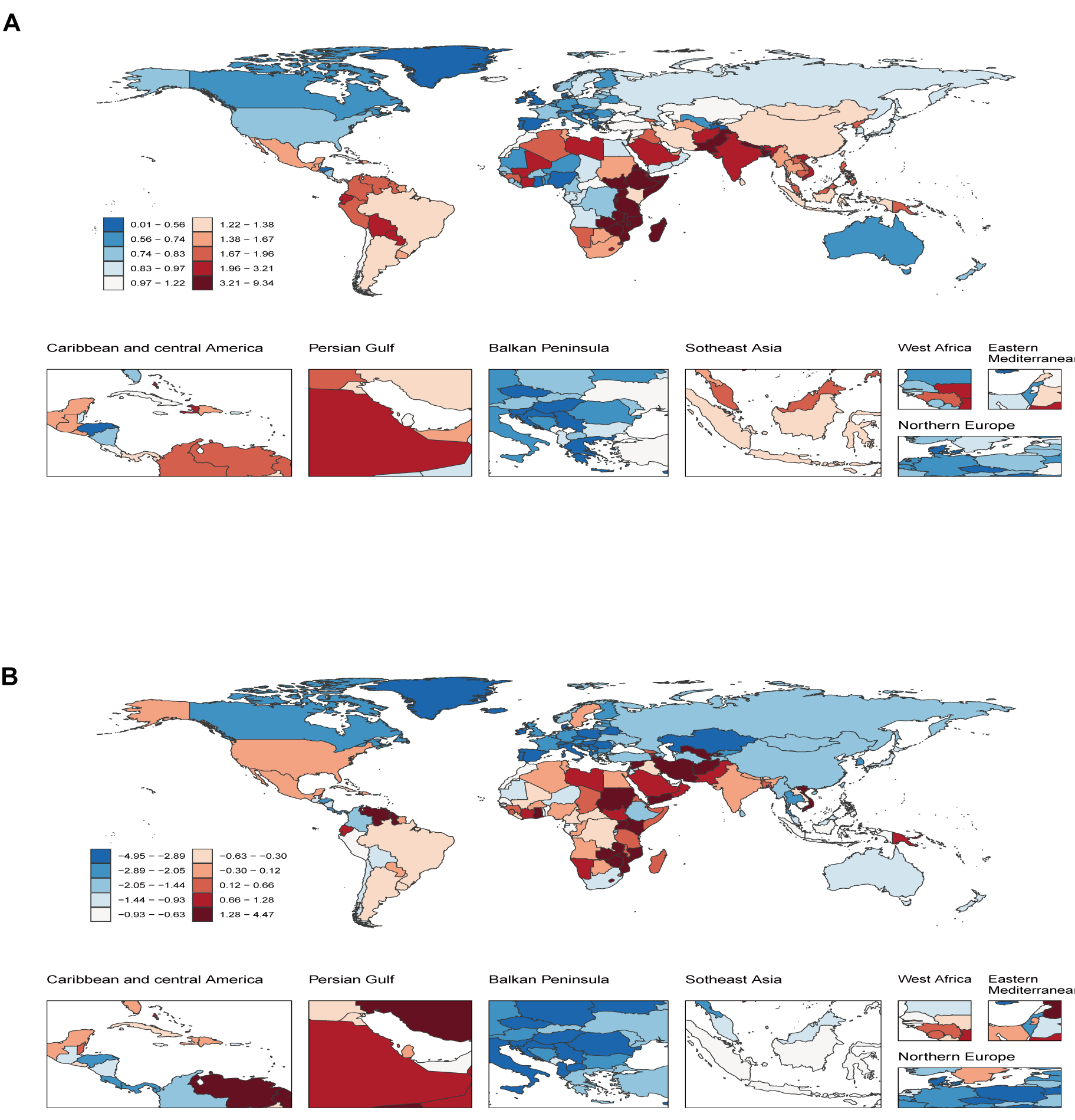

Supplement: Supplementary file 1 [file DataSheet1.zip › Additional files/Additional file1(FigureS1-S12)/FigureS5.tif]

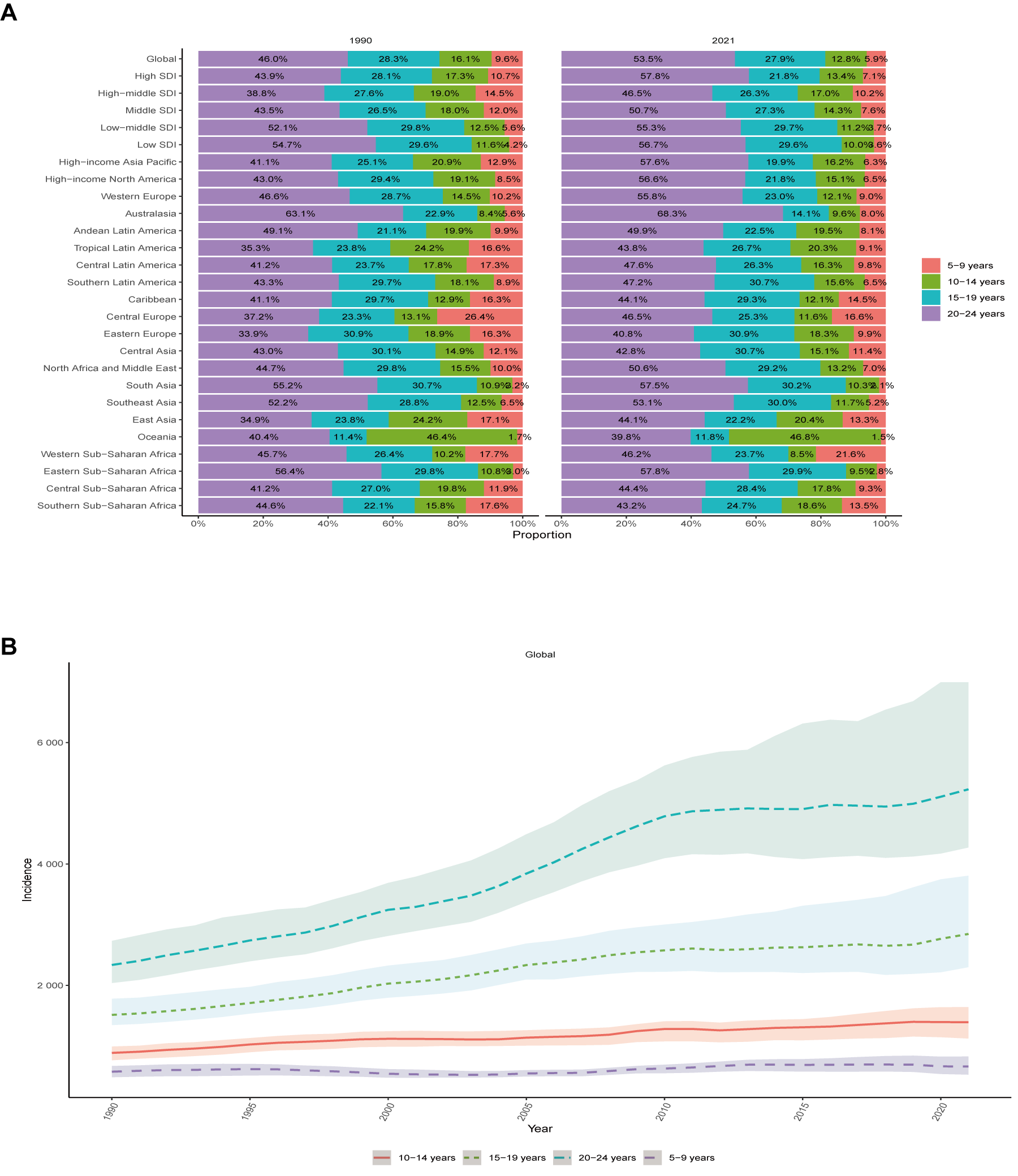

Supplement: Supplementary file 1 [file DataSheet1.zip › Additional files/Additional file1(FigureS1-S12)/FigureS6.tif]

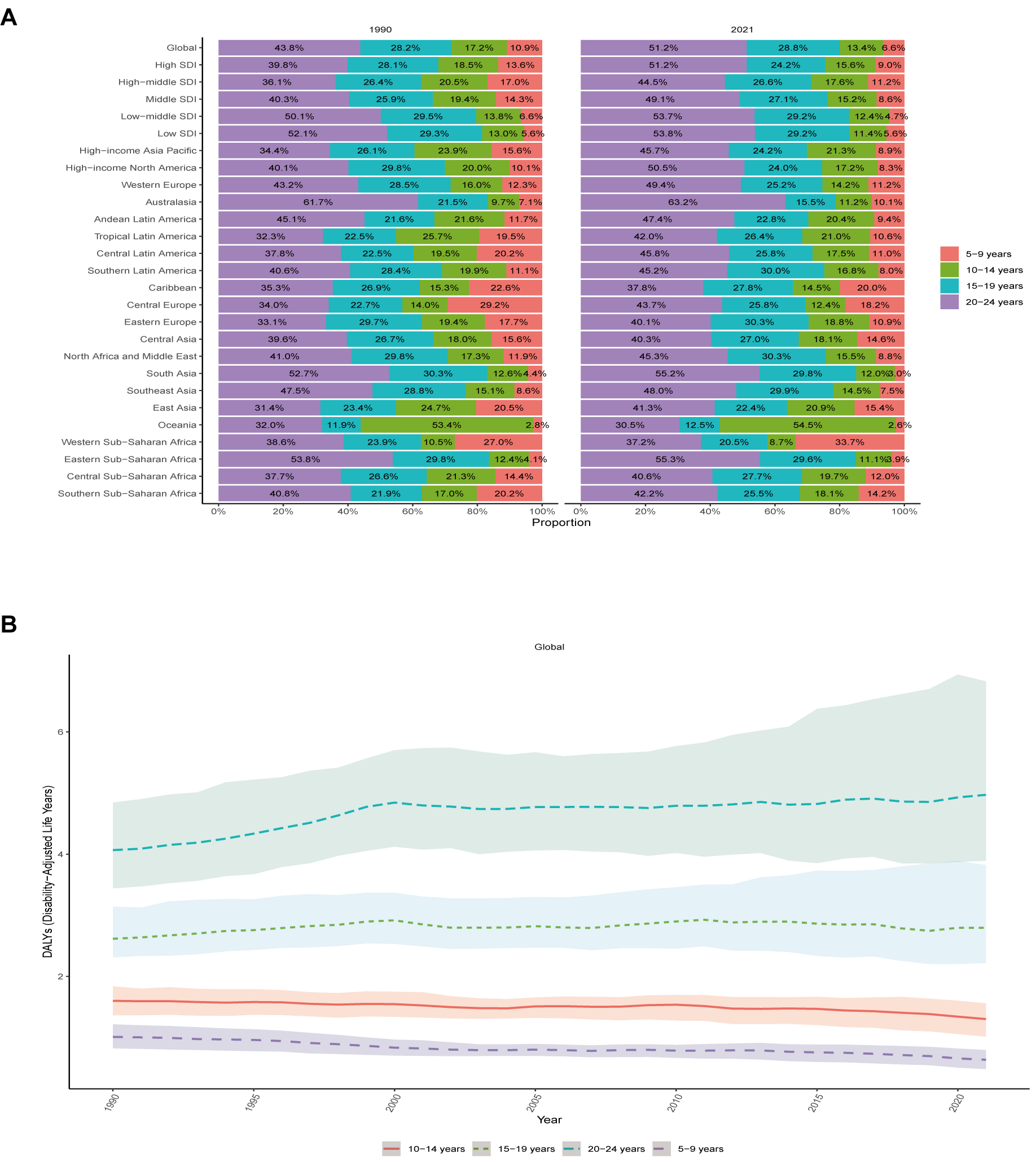

Supplement: Supplementary file 1 [file DataSheet1.zip › Additional files/Additional file1(FigureS1-S12)/FigureS7.tif]

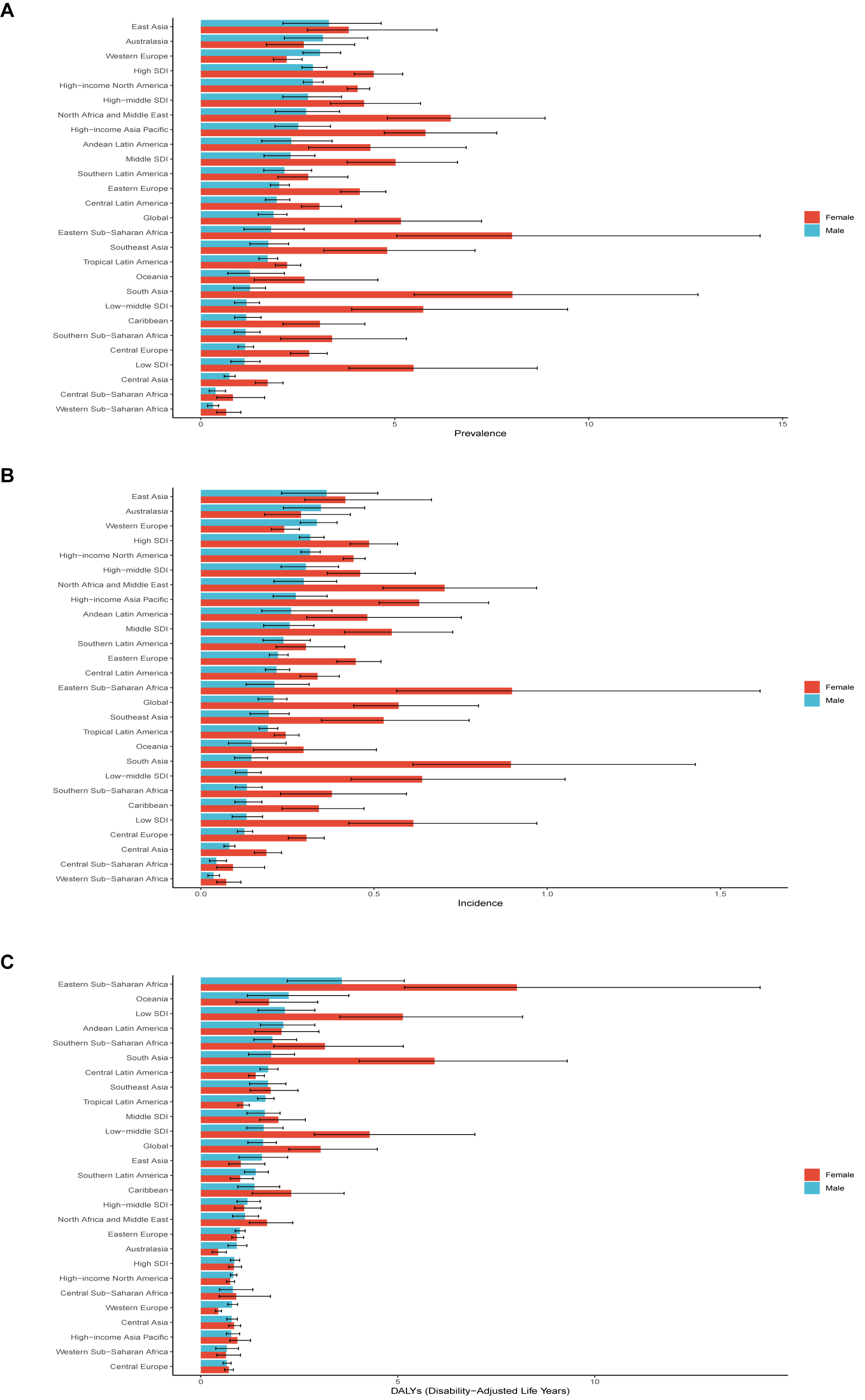

Supplement: Supplementary file 1 [file DataSheet1.zip › Additional files/Additional file1(FigureS1-S12)/FigureS8.tif]

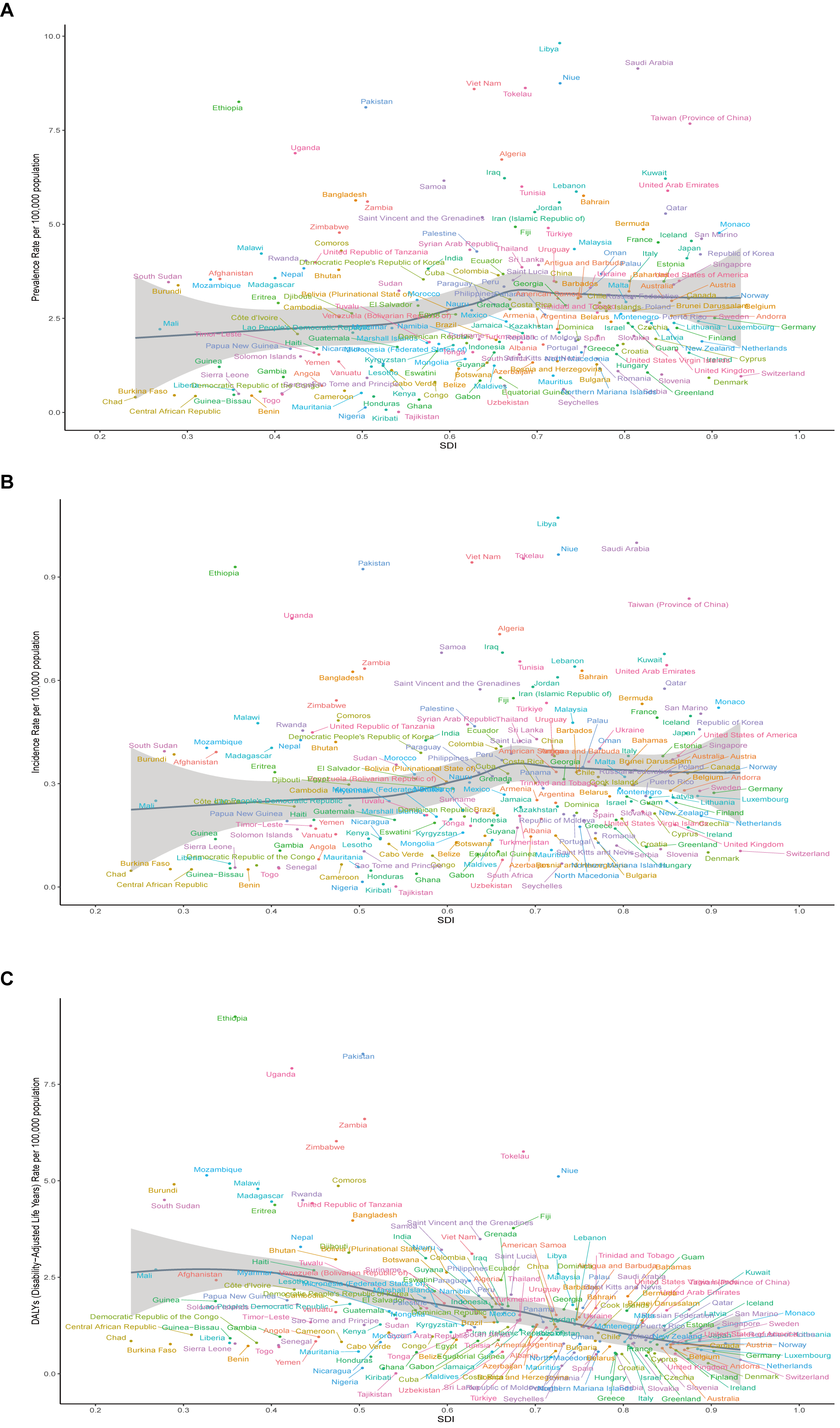

Supplement: Supplementary file 1 [file DataSheet1.zip › Additional files/Additional file1(FigureS1-S12)/FigureS9.tif]
